# Cyanobacteria and Microalgae as Promising Sustainable Sources of Bioactive Phytochemicals for Nutraceutical and Pharmaceutical Applications

**DOI:** 10.3390/ijms27156764

**Published:** 2026-07-28

**Authors:** Zouhair Essahli, Tarik Sabour, Yassine Boutkida, Karim Lyamlouli, Abdellah Oukarroum, Tarik Aanniz, Nawal Merghoub, Abdelhakim Bouyahya, Imane Chamkhi

**Affiliations:** 1 Geo-Biodiversity and Natural Patrimony Laboratory (GeoBio), Geophysics, Natural Patrimony Research Center (GEOPAC), Scientific Institute, Mohammed V University in Rabat, BP 703, Rabat 10106, Morocco; zouhair.essahli@um5r.ac.ma (Z.E.); tarik.sabour@um5r.ac.ma (T.S.); yassineboutkida@gmail.com (Y.B.); 2 AgroBiosciences, College for Sustainable Agriculture and Environmental Sciences, Mohammed VI Polytechnic University, Hay Moulay Rachid, Ben Guerir 43150, Morocco; karim.lyamlouli@um6p.ma (K.L.); abdallah.oukarroum@um6p.ma (A.O.); 3 Laboratory of Human Pathologies Biology, Faculty of Sciences, Mohammed V University in Rabat, BP 1014, Rabat 10106, Morocco; a.bouyahya@um5r.ac.ma; 4 Medical Biotechnology Laboratory, Rabat Medical & Pharmacy School, Mohammed V University in Rabat, BP 1014, Rabat 10106, Morocco; 5 Plant & Microbial Biotechnology Center, Moroccan Foundation for Advanced Science, Innovation & Research (MASCIR), Mohammed VI Polytechnic University (UM6P), Lot 660, Hay Moulay Rachid, Ben Guerir 43150, Morocco; nawal.merghoub@um6p.ma

**Keywords:** secondary metabolites, bioactive compounds, Cyanobacteria, microalgae, biological effect

## Abstract

This review aims to assess and synthesize the recent scientific literature on bioactive phytochemicals derived from Cyanobacteria and microalgae, with a focus on their pharmaceutical and nutraceutical applications. A total of 837 articles published over the past seven years were retrieved from multiple databases, including studies reporting antioxidant, anti-inflammatory, antimicrobial, and other pharmacological and nutritional properties. After applying defined inclusion and exclusion criteria, 86 studies were selected for detailed analysis. Among these, 28% focused on nutritional and dietary applications, 26% on antioxidant activities, 22% on other pharmacological potentials, 13% on antimicrobial effects, and 11% on anti-inflammatory properties. The most frequently studied genera were *Arthrospira*/*Limnospira* (Cyanobacteria) (29 articles), followed by *Chlorella* (Chlorophyta) (13), *Nannochloropsis* (10), and *Phaeodactylum* (Bacillariophyceae) (8). Regarding the types of products studied, crude extracts or biomass were the most common (68.96%), followed by pigments (10.34%), lipids (8.04%), proteins (6.89%), and polysaccharides (5.74%). In terms of methodology, 72% of the studies employed in vitro approaches, with 42% using non-cellular assays and 30% using cellular assays, while in vivo and in silico approaches were used in 23% and 5% of studies, respectively. The available literature indicates that Cyanobacteria- and microalgae-based products are a promising source of bioactive compounds for pharmaceutical and nutraceutical applications. However, as most of the current evidence remains preliminary and derived largely from in vitro studies, these findings should be regarded as an early indication of potential that requires further in vivo and clinical validation.

## 1. Introduction

In recent years, there has been a growing global demand for novel compounds for both therapeutic and nutritional purposes [1]. This surge is driven by the increasing prevalence of chronic diseases, antibiotic resistance, and the need for sustainable food sources to meet the nutritional demands of a growing population [2].

People suffering from various pathologies often rely heavily on synthetic medications and treatments that only provide symptom control and may lead to undesirable side effects, while health-conscious individuals are seeking natural, safe, and effective alternatives to support their well-being [3]. Among the most promising natural resources are marine-sourced compounds, particularly those derived from microalgae and Cyanobacteria [4]. The term “*microalgae*” refers to microscopic, unicellular eukaryotic algae that may live individually or associate in chains or colonies. Together with Cyanobacteria, they constitute the first link of the aquatic trophic chain (phytoplankton) and have played a key role in the progressive oxygenation in the Earth’s atmosphere over time [5].

Due to their remarkable biodiversity and their capacity to produce unique secondary metabolites, they are emerging as a valuable source of diverse bioactive molecules [6]. Depending on their taxonomic group, microalgae and Cyanobacteria are particularly rich in lipids (up to 50%), proteins (up to 70%), polysaccharides, pigments, minerals, and various bioactive compounds such as polyphenols [7,8]. This biochemical richness makes microalgae highly attractive for a wide range of applications: (1) human and animal nutrition (dietary supplements, natural colorants, texturizing agents, etc.), (2) cosmetics (sources of antioxidant molecules, UV filters, moisturizing and anti-aging agents, etc.), (3) energy production (biofuels), and (4) other fields such as industrial CO_2_ capture. The microalgae sector has shown consistent commercial growth, although reported market valuations vary widely. However, global market estimates placed the microalgae market at approximately USD 3.4 billion in 2020, with projected growth to around USD 4.6 billion by 2027 [9,10]. Other analyses report broadly similar upward trajectories but differ considerably in absolute terms, from roughly USD 1 billion to over USD 13 billion, depending on market scope, segment definition, and forecasting methodology [11]. This growth is driven by strong demand from the dietary supplement and pharmaceutical sectors.

These photosynthetic microorganisms are recognized for their rapid growth, high adaptability, and remarkable ability to synthesize a diverse array of bioactive molecules [12]. For over six decades, microalgae and Cyanobacteria have served as important biological sources for the development of functional foods, nutraceuticals, and therapeutic agents [4]. In this context, it should be noted that the terms nutraceutical and pharmaceutical should not be considered under the same definition framework. According to the U.S. Food and Drug Administration (FDA), pharmaceuticals are regulated under the category of drugs, defined as medicinal products intended for the diagnosis, treatment, mitigation, or prevention of diseases and subject to rigorous evaluation of their safety, efficacy, and quality [13]. In contrast, nutraceuticals, often associated with the concept of dietary supplements, are generally defined as food- or natural-product-derived substances that provide health benefits beyond basic nutrition and are primarily intended to support health maintenance and overall well-being [14,15]. These products are intended to supplement the diet and are distinct from conventional foods. However, under FDA regulations, any product intended to diagnose, treat, cure, or prevent disease is classified as a drug, regardless of whether it is labeled as a dietary supplement, and is therefore subject to drug regulatory requirements [16]. Although these categories are traditionally distinguished by their intended use and regulatory requirements, the distinction is not always clear, as several bioactive compounds derived from microalgae and Cyanobacteria exhibit both health-promoting and therapeutic properties. Consequently, the classification of these compounds may depend not only on their biological activities but also on factors such as dosage, intended application, approved health claims, and the regulatory framework under which they are marketed and utilized. Bioactive compounds such as phycocyanin, astaxanthin, β-carotene, and polyunsaturated fatty acids have been reported to exhibit potent antioxidant, anti-inflammatory, antidiabetic, and anti-obesity properties, suggesting promising results in the prevention of inflammatory diseases and cardiovascular disorders [17,18,19,20]. For instance, carotenoid-rich fractions of *Dunaliella salina* attenuated inflammation-associated cardiac dysfunction in obese rats [20], and macular pigments (lutein and zeaxanthin) from *Chlorella* sp. alleviated diabetic and atherosclerotic damage in mice [19], while extracts of the green microalga *Chlamydomonas agloeformis* reduced oxidative and inflammatory markers in vascular endothelial cells in vitro [17]. Other compounds are also derived from a wide range of cyanobacterial and microalgal species such as phycocyanin extracted from *Limnospira platensis* (formerly *Spirulina platensis*) and *Desmonostoc alborzicum* (Cyanobacteria), which have demonstrated antimicrobial and anticancer properties, largely attributed to their strong antioxidant and anti-inflammatory activities [21,22]. Polysaccharides and lipids derived from microalgae and Cyanobacteria have also demonstrated anti-inflammatory properties; for example, the exopolysaccharide of *Chlorella vulgaris* has demonstrated potent anti-inflammatory properties, notably through the elevation of IL-12 and IFN-γ levels, exerting an anti-remodeling effect in a guinea-pig model of ovalbumin-induced asthma [23]. On the other hand, the total polar lipid extract of the freshwater cyanobacterium *Gloeothece* sp. has shown anti-inflammatory and antioxidant activity in vitro, inhibiting COX-2 activity (≈58% reduction in prostaglandin production at 10 μg mL^−1^) and scavenging superoxide (O_2_·^−^ and nitric oxide (·NO) radicals [6].

Despite numerous reports in the literature demonstrating the bioactivities of compounds derived from Cyanobacteria and microalgae, the overall evidence supporting their potential as sustainable sources of bioactive molecules for nutraceutical and pharmaceutical applications remains fragmented. Although these microorganisms exhibit highly promising biochemical profiles, most existing studies focus on isolated biological activities without integrating broader aspects of chemical composition, extraction methodologies, and underlying mechanisms of action. This lack of integrative analysis limits a comprehensive understanding of how variations in chemical composition, metabolite profiles, and extraction methods directly influence the observed biological activities and therapeutic potential of bioactive compounds derived from different Cyanobacterial and microalgal strains.

To address these gaps, it is essential to consolidate the scattered body of knowledge, assess real-world applicability, and evaluate the scalability and translational potential of these bioactive compounds. Moreover, the present work provides, to the best of our knowledge, the first review that integrates both pharmaceutical and nutraceutical applications of bioactive compounds derived from Cyanobacteria and microalgae within a unified and comprehensive analytical framework. Whereas earlier reviews have documented the therapeutic and nutritional properties of these microorganisms [24,25], they have generally addressed these aspects separately rather than in an integrated manner. By combining these dimensions, the present review aims to offer a consolidated and multidimensional view of the scientific literature in order to compile, analyze, and integrate recent findings on Cyanobacteria- and microalgae-derived bioactive compounds and to highlight their biological properties relevant to pharmaceutical and nutraceutical applications that may guide future research directions in the field.

## 2. Methods

### 2.1. Data Sources and Search Strategy

A comprehensive literature search was performed using three databases: PubMed^®^ (National Library of Medicine, Bethesda, MD, USA), ScienceDirect^®^ (Elsevier B.V., Amsterdam, The Netherlands), and Scopus^®^ (Elsevier B.V., Amsterdam, The Netherlands). In each database, the search terms were applied to the title, abstract, and keywords fields. The search strings combined terms related to the organisms (“Cyanobacteria,” “microalgae,” “blue-green algae”), the bioactive compounds (“bioactive compounds,” “natural products”), and their applications (“pharmaceuticals,” “therapeutics,” “medicine,” “nutraceuticals,” “functional foods”), together with terms for the biological activities of interest (“antioxidant,” “anti-inflammatory,” “antimicrobial,” “anti-cancer”). Terms were combined using the Boolean operators AND and OR. The search strings were adapted to the specific interface and Boolean requirements of each database; the exact string used in each is reported below. The search terms were used as plain keywords, without wildcard truncation or database-specific controlled vocabulary. In the second stage, a screening process was conducted where, after removing duplicates, articles were screened based on their titles and abstracts to exclude studies that did not meet the inclusion criteria. The final selection of articles was made after full-text screening to ensure they satisfied the inclusion criteria. The selection was restricted to articles published in English, and databases were accessed within a seven-year period from 1 January 2018 to 31 December 2024.

This review was conducted in accordance with scoping/narrative review guideline. However, the review protocol was not registered in a public database.

#### 2.1.1. ScienceDirect

(“Cyanobacteria” OR “microalgae”) AND (“bioactive compounds” OR “natural products”) AND (“pharmaceuticals” OR “medicine”) AND (“antioxidant” OR “anti-inflammatory” OR “antimicrobial” OR “anti-cancer”) AND (“sustainability” OR “nutraceuticals”) AND “experimental”.

#### 2.1.2. Scopus

(“Cyanobacteria” OR “microalgae” OR “blue-green algae”) AND (“bioactive compounds” OR “natural products”) AND (“pharmaceuticals” OR “therapeutics” OR “medicine”) AND (“nutraceuticals” OR “functional foods”) AND (“experimental” OR “in vitro” OR “in vivo” OR “study” OR “results”).

#### 2.1.3. PubMed

(Cyanobacteria OR microalgae OR blue-green algae) AND (bioactive compounds OR natural products) AND (pharmaceuticals OR therapeutics OR medicine) AND (sustainability OR green technology) AND (nutraceuticals OR functional foods) AND (experimental OR study OR in vitro OR in vivo OR results).

### 2.2. Inclusion/Exclusion Criteria and Selection Process

Two independent reviewers conducted the selection of relevant studies. The review included original research articles from the scientific literature reporting antioxidant, anti-inflammatory, antimicrobial, anticancer, or other pharmacological or nutritional properties of microalgae, Cyanobacteria, or their extracts/biomass, with relevance for incorporation into pharmaceutical and nutraceutical applications. Exclusion criteria were: (i) studies focused on topics unrelated to nutraceuticals or pharmaceuticals (e.g., biofuel production, environmental remediation, or basic taxonomy without applied context); (ii) research centered on organisms other than Cyanobacteria or microalgae (e.g., higher plants, fungi, macroalgae, or unrelated microorganisms); (iii) non-primary studies, including editorials, letters to the editor, literature reviews, commentaries, and conference papers; and (iv) articles published prior to 2018, as this review focused on the last seven years (2018–2024).

### 2.3. Analysis of Co-Occurrence of the Selected Keywords

The co-occurrence of keywords identified in the literature was analyzed using VOSviewer software (version 1.6.20; Centre for Science and Technology Studies (CWTS), Leiden University, Leiden, The Netherlands). A network visualization map was generated to cluster keywords into thematic groups and illustrate their interrelationships. Connection strength was color-coded to indicate similarity, with lighter hues representing weaker associations between keywords. The size of nodes (circles) and their labels within each cluster correspond to their co-occurrence frequency, while the proximity of nodes and the thickness of connecting lines indicate the degree of relatedness between terms. A minimum occurrence threshold of four mentions per keyword was applied to ensure analytical relevance, filtering out infrequent terms from the visualization.

### 2.4. Extraction and Definition of Data

From the selected papers, data were categorized based on the following criteria: (i) characteristics of the selected studies, (ii) genera and species studied, (iii) microalgae- and Cyanobacteria-based products, (iv) study models, (v) pharmacological applications, and (vi) nutraceutical applications. Additionally, studies exploring pharmacological applications were further subcategorized according to the type of therapeutic application, including antioxidant potential, anti-inflammatory properties, antimicrobial activity, and other pharmacological effects. Similarly, nutraceutical properties were classified based on the type of bioactive compound and its corresponding nutraceutical value.

## 3. Results

**Figure 1:**
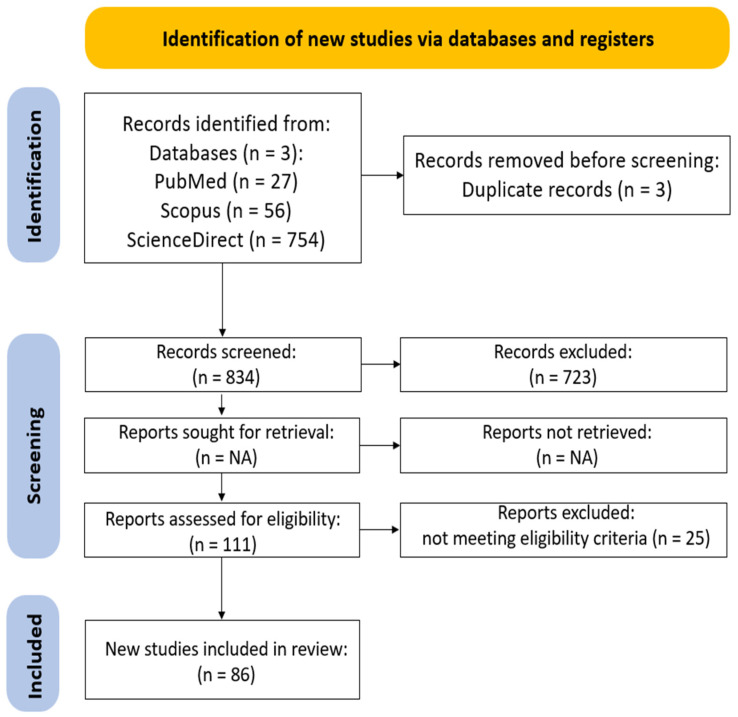
Overview of the study selection process flow diagram.

The literature search, following the criteria outlined in Section 2, identified 837 potentially relevant articles across three databases: 754 in ScienceDirect^®^, 56 in Scopus^®^, and 27 in PubMed^®^. After removing duplicates (*n* = 3), a total of 834 records were processed. These papers underwent an initial screening based on titles and abstracts, during which 723 records were excluded for not meeting the inclusion criteria. The remaining 111 records were then assessed for eligibility through full-text screening, after which 25 were excluded. Exclusions at this stage were attributable to the predefined criteria (Section 2.2), namely: applications unrelated to pharmaceutical or nutraceutical use, a focus on organisms other than Cyanobacteria or microalgae, and non-primary study types (e.g., reviews, conference papers), resulting in a final selection of 86 studies for inclusion in this review (Figure 1). Data and key findings from the 86 selected studies are summarized in two comprehensive tables: one table compiles data on pharmacological applications, including antioxidant potential, anti-inflammatory effects, antimicrobial activity, and other therapeutic properties (Table 1), while the second table presents data on nutraceutical properties, highlighting the bioactive compounds studied and their reported health benefits (Table 2).

### 3.1. Keyword Frequency and Co-Occurrence Mapping

**Figure 2:**
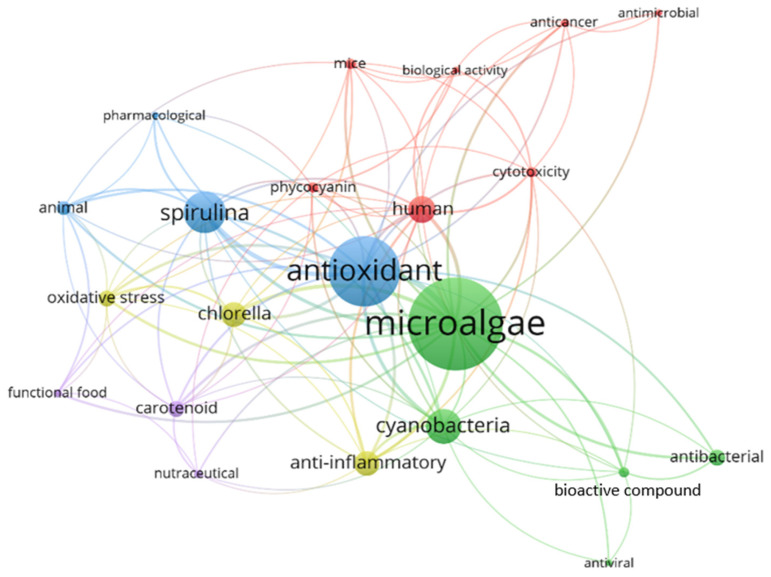
Network map of keyword co-occurrences of included papers from the years 2018–2024, divided into clusters using VOSviewer software (version 1.6.20).

Co-occurrence analysis identifies relationships and associations between keywords within a dataset, providing insights into primary research topics within a specific field [98]. In this review, co-occurrence analysis was employed to confirm the relevance of selected keywords for the topics under investigation. From 423 keywords extracted across 86 articles, 21 keywords met the threshold of three occurrences. These keywords were analyzed for co-occurrence frequency and total link strength, which indicates the total number of cited references shared between items. The analysis results were visualized as a network (Figure 2), grouping the 21 keywords into five distinct clusters. Cluster 1 (red) consisted of 7 keywords: “anticancer”, “antimicrobial”, “biological activity”, “cytotoxicity”, “human”, “mice”, and “phycocyanin”. Phycocyanin, a bioactive pigment-protein complex from Cyanobacteria, appears central to this cluster. Cluster 2 (green) comprised 5 keywords: “antibacterial”, “antiviral”, “bioactive compound”, “Cyanobacteria” and “microalgae”. Cluster 3 (blue) included 4 keywords: “animal”, “antioxidant”, “pharmacological” and “spirulina”, a widely studied cyanobacterium. The inclusion of “animal” indicates frequent use of in vivo models to assess compound efficacy in some of the included studies. Cluster 4 (yellow) comprised 3 keywords: “anti-inflammatory”, “oxidative stress”, and “Chlorella”. Cluster 5 (purple) included 3 keywords: “carotenoids”, “functional food” and “nutraceutical”.

### 3.2. Study Types

**Figure 3:**
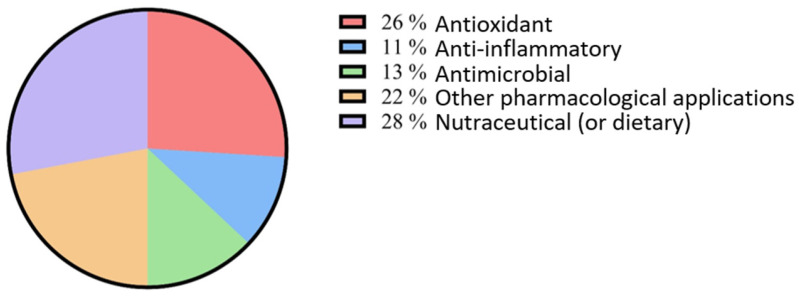
Distribution and frequency of selected papers from the years 2018–2024. The number of papers in each defined category was counted and expressed as a percentage.

The selected studies that met the inclusion criteria (see Section 2.2) focus on pharmaceutical applications, such as antioxidant, anti-inflammatory, antimicrobial activities, and other pharmacological effects, as well as nutritional and dietary applications. As shown in Figure 3, 26% of the studies reported antioxidant properties and 28% nutrition and dietary applications, 22% explored other pharmacological applications, 13% investigated antimicrobial activity, and 11% examined anti-inflammatory potential. These results indicate that antioxidant activity and nutritional and dietary properties have been the most extensively studied in microalgae and Cyanobacteria extracts. In contrast, anti-inflammatory and antimicrobial potentials remain underexplored, highlighting a research gap that could be addressed in future studies to further explore their pharmaceutical potential. It should be noted, however, that these percentages represent the distribution of the included studies across activity and application categories—that is, the relative research attention each category has received—and do not indicate the strength, quality, reproducibility, or clinical relevance of the corresponding evidence.

### 3.3. Genera Studied

**Figure 4:**
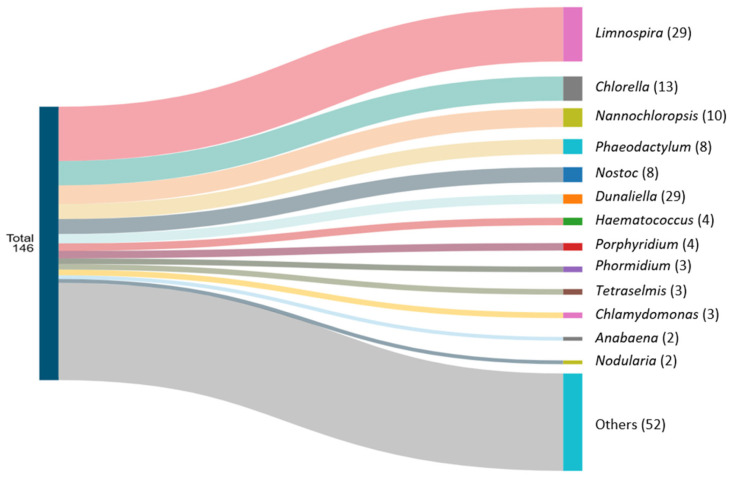
The frequency of organisms within each genus reported in the included study articles from the years 2018–2024.

The frequency of genera occurrences in the papers included in this review reveals a distinct research focus (Figure 4). *Limnospira* (formerly *Arthrospira*) (29) emerges as the most extensively studied genus, followed by moderately represented genera, such as *Chlorella* (13), *Nannochloropsis* (10) and *Phaeodactylum* (8). In contrast, lower-frequency genera like *Dunaliella* (5) and *Haematococcus* (4) receive more limited attention. The substantial “Others” category (52) highlights the broad taxonomic diversity within the literature, including numerous genera that, while individually less studied, collectively account for nearly half of the occurrences.

### 3.4. Cyanobacteria- and Microalgae-Based Products

**Figure 5:**
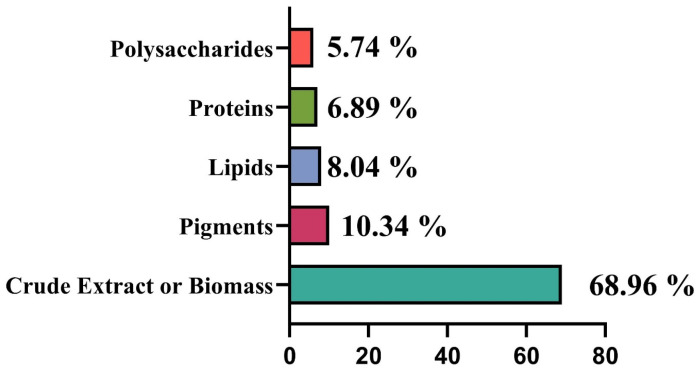
The frequency of microalgae- and Cyanobacteria-based products reported in the included study articles from the years 2018–2024. The number of papers of each defined category was counted and expressed as a percentage.

The products derived from Cyanobacteria and microalgae analyzed in the included studies were categorized into crude extracts or biomass (powder), proteins, pigments, lipids, and polysaccharides. Crude extracts or biomass (68.96%) represented the most frequently studied category, followed by pigments (10.34%), lipids (8.04%), proteins (6.89%), and polysaccharides (5.74%) (Figure 5).

### 3.5. Study Models

**Figure 6:**
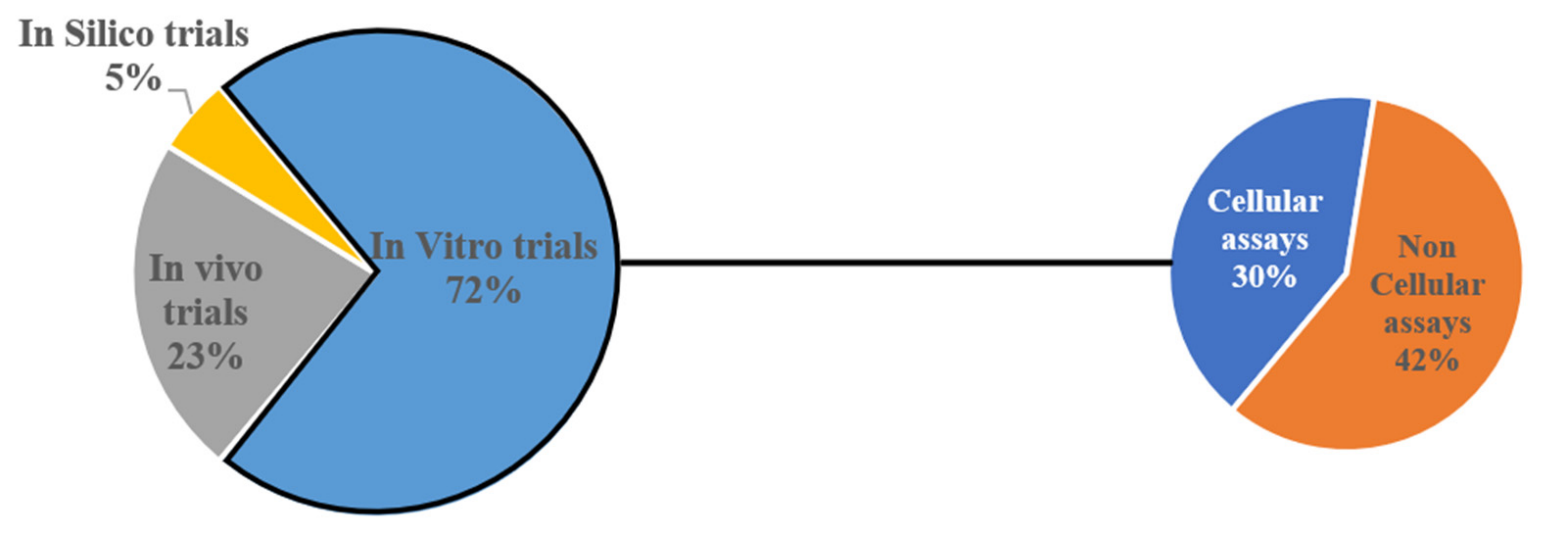
The frequency of study models reported in the included study articles from the years 2018–2024. The number of papers of each defined category was counted and expressed as a percentage.

Overall, in vitro assays constitute the predominant methodology, representing 72% of the included studies (Figure 6). These assays are further categorized as cellular assays (30%) and non-cellular assays (42%). In vivo trials account for 23% of the studies, while in silico approaches are the least common methodology, comprising only 5% of the reviewed papers. Notably, some studies employed multiple methodologies; in such cases, each methodology was counted separately to reflect the full scope of experimental approaches adopted.

## 4. Discussion

The potential of new natural sources for pharmaceutical and nutraceutical applications is attracting growing interest. While most evaluations have traditionally focused on terrestrial organisms, increasing attention is being given to aquatic organisms, particularly microalgae and Cyanobacteria, due to their remarkable biodiversity and their capacity to produce unique secondary metabolites [72]. These compounds are emerging as a valuable source of diverse bioactive molecules. Microalgae and Cyanobacteria are thus increasingly regarded as candidate sources for the future development of pharmaceutical and nutraceutical products [82], thanks to their bioactive molecules with a wide range of properties, including antioxidant, anti-inflammatory, antimicrobial, and antitoxic effects, among others [26,84]. However, as most of the current evidence remains preliminary and derived largely from in vitro studies, these findings should be regarded as an early indication of potential that requires further in vivo and clinical validation.

### 4.1. Genera

Among the species included in this review, *Limnospira platensis* (formerly known as *Arthrospira*/*Spirulina*) is the cyanobacterium most frequently used. It has attracted considerable attention owing to its rich content of bioactive molecules and natural pigments, including carotenoids (β-carotene and zeaxanthin), chlorophyll, phycocyanin, and phycobiliproteins, which are regarded as the most valuable photosynthetic pigments of this species [55]. Currently, *Limnospira* accounts for approximately 60% of global microalgal production [26].

*Chlorella* and *Nannochloropsis* are two further Chlorophyta genera that were widely represented across the various research areas covered by the included studies [48,59]. It has been reported that the microalgae most cultivated in Europe, measured by the number of companies active in biotechnology applications, are *Chlorella* spp., *Nannochloropsis* spp., and *Haematococcus*, together with *Limnospira*. This suggests that the frequent appearance of these genera in the literature may partly reflect their established commercial cultivation and large-scale production, as well as historical research focus, rather than comparative biological promise alone. These genera are also among the most extensively exploited worldwide over recent decades in the pharmaceutical and food industries, where they serve as dietary supplements providing vitamins, highly digestible proteins, polysaccharides, and lipids (e.g., PUFAs) [99].

Beyond the widely used *Limnospira*, other cyanobacterial strains such as *Nodularia* sp., *Phormidium* sp., *Nostoc* sp., and *Anabaena* sp. could therefore represent promising yet underexplored sources of antioxidant and anti-inflammatory compounds for biotechnological applications in functional foods, nutraceuticals, and pharmaceuticals [26].

### 4.2. Microalgae- and Cyanobacteria-Based Products

Extraction is a crucial step following the production of sufficient algal biomass, as the choice of extraction technique and solvents can significantly impact the efficiency and selectivity of bioactive compound recovery [6]. In this review, the included studies were categorized into five main types of extracted products from microalgae and Cyanobacteria: crude extracts or biomass, pigments, lipids, proteins, and polysaccharides.

#### 4.2.1. Crude Extract or Biomass

Crude extracts are typically obtained by treating biomass with appropriate solvents, followed by solvent evaporation. Several factors influence extraction efficiency, including time, temperature, and the degree of biomass fragmentation. Additionally, the solvent type plays a key role in determining the chemical composition of the extract. Commonly used solvents include ethanol, methanol, hexane, ethyl acetate, acetone, and water, each selected based on the polarity of the targeted compounds [27,30,100]. The frequent use of crude extracts or biomass (68.96%) among the studies included in this review can be attributed to the low cost and simplicity of the methods employed, which do not require compound purification; this predominance therefore reflects practical and methodological convenience rather than any greater clinical or developmental potential of the crude extracts relative to purified fractions. Biomass may be used in dry or wet form or subjected to various physical treatments such as freezing/thawing, high-pressure, ultrasound-assisted extraction or sonication, grinding, or microwave processing [3,12,41]. Crude extracts and biomass have demonstrated a range of biological activities, although the specific outcomes vary markedly with experimental conditions. For example, methanolic extracts of *Micractinium* sp. showed strong antioxidant capacity, with 7.72 and 93.80 µmol trolox equivalents g^−1^ DW based on DPPH and FRAP assays, respectively [35]. Comparable radical-scavenging activity was reported for crude extracts of *Chlorella vulgaris* and *Scenedesmus incrassatulus* obtained with natural deep eutectic solvents (DPPH IC_50_ of 5.58 and 3.98 mg g^−1^) [44], while the different solvents, assays, and units used across these studies mean that their results cannot be directly compared and show how much the extraction conditions can affect the activity observed. Anti-inflammatory activity has also been reported: For example, the extract from *Auxenochlorella pyrenoidosa* (formerly *Chlorella pyrenoidosa*) significantly reduced inflammatory cytokines (IL-6, IL-1β, TNF-α) and macrophage infiltration in colon tissue, suggesting its potential as a therapeutic agent for ulcerative colitis [3], while a broader screening of ten cyanobacterial strains reported anti-inflammatory and antioxidant activity across crude extracts but with substantial variation between strains [26], underscoring that such effects are potentially strain-dependent rather than uniform across taxa.

From a nutritional and dietary perspective, crude extracts and biomass from Cyanobacteria and microalgae contain a diverse array of bioactive compounds with promising applications. For instance, ethanolic extracts from *Nostoc commune*, rich in phytochemicals such as polyphenols, flavonoids, and terpenoids, significantly reduced body weight (13.5%), fat tissue (13.3%), and serum levels of FFA (19.4%), TG (14.2%), TC (11.8%), and LDL-C (16.4%) in rats [75].

#### 4.2.2. Pigments

Pigments are naturally occurring compounds found in both terrestrial and aquatic organisms, with certain types such as chlorophyll c, phycobiliproteins, and specific carotenoids (e.g., β-carotene, α-carotene, and various xanthophylls) being almost exclusively associated with aquatic microorganisms like Cyanobacteria and microalgae [18,100]. Pigments such as carotenoids are primarily derived from isopentenyl pyrophosphate (IPP), which is synthesized through two distinct metabolic routes: the mevalonate (MVA) pathway and the non-mevalonate pathway, also known as the 1-deoxy-D-xylulose 5-phosphate/2-C-methylerythritol 4-phosphate (DOXP/MEP) pathway, in microalgae. In contrast, Cyanobacteria synthesize carotenoids and other terpenoids exclusively via the DOXP/MEP pathway, as they lack the mevalonate (MVA) pathway [101]. Pigments are of growing interest due to their wide spectrum of biological activities, particularly their antioxidant and anti-inflammatory properties [46]. These reported activities are not uniformly beneficial; however, some findings warrant cautious interpretation. For instance, ref. [18] examined the effects of phycoerythrin and phycocyanin from *Nostoc* sp. and *Limnospira* sp. on human skin fibroblasts (CCD-966SK) and reported a concentration-dependent dual effect: At low concentrations, the pigments elicited no IL-6 or TNF-α release, whereas at higher concentrations, they significantly increased these pro-inflammatory cytokines and reduced cell viability, with marked necrosis, cytoskeletal disruption, declining collagen I/II levels, and elevated ROS/MDA. The pigments were therefore anti-inflammatory only within a narrow low-dose window and became pro-inflammatory and cytotoxic to normal fibroblasts as the dose increased, underscoring the need to distinguish genuine anti-inflammatory activity from dose-dependent cytotoxicity when appraising pigment bioactivity. By contrast, more robust anti-inflammatory activity has been reported for C-phycocyanin, which reached inhibition values of up to 98.76 ± 0.065% [21].

In terms of antioxidant activity, astaxanthin extracted from *Haematococcus* sp. showed strong antioxidant potential using the ABTS assay [37], and comparable carotenoid-associated antioxidant activity has been reported for pigment-rich extracts of other cyanobacteria and microalgae [32,33]; the degree of activity nonetheless varied with pigment composition, extraction method, and the assay applied, indicating that pigment class and processing are key determinants of the measured effect. As these findings were obtained using different assays and units, they indicate a shared qualitative antioxidant capacity across these pigment-rich extracts rather than a comparable ranking of potency.

Beyond antioxidant and anti-inflammatory roles, pigments such as lutein and zeaxanthin, which are macular pigments derived from *Chlorella* sp., have shown therapeutic potential in alleviating symptoms of type 2 diabetes mellitus in ApoE^−^/^−^ diabetic mice [19]. C-phycocyanin from *Limnospira platensis* (formerly *Spirulina platensis*) also demonstrated significant anticancer activity, reducing cancer cell viability by up to 74% at the highest tested concentration (50 µg) and 63% at the lowest (6.25 µg) [21]. From a nutritional and functional food perspective, a diet enriched with *Tisochrysis lutea* (Coccolithophyceae) led to improved digestibility and increased HDL cholesterol (*p* < 0.05), highlighting its potential as a source of bioactive compounds for metabolic health [88].

#### 4.2.3. Lipids

Microalgae and Cyanobacteria are recognized as sustainable sources of essential and bioactive lipids, offering a promising alternative to conventional lipid sources. They exhibit considerable diversity in their fatty acid profiles and abundances, enhancing their biotechnological potential [102]. The lipid fraction of these microorganisms typically consists of monogalactosyl diacylglycerol (MGDG, ~40%), digalactosyl diacylglycerol (DGDG, ~32%), sulfoquinovosyl diacylglycerol (SQDG, ~15%), and phosphatidylglycerol (PG, ~13%) [6]. These lipid classes are attracting increasing interest due to their documented antioxidant, anti-inflammatory, and antimicrobial properties [6,33,38,45,47,52]. Ref. [86] reported that the Cyanobacterial and microalgal strains investigated in their study produced 14 types of fatty acids, with chain lengths ranging from C14 to C18, including both odd- and even-numbered carbon atoms and varying degrees of unsaturation. Oleic acid was found to be the most abundant. Moreover, lipid extracts from *Gloeothece* sp. displayed dose-dependent antioxidant activity against superoxide (O_2_·^−^) and nitric oxide (·NO) radicals, with IC_50_ values of 939.8 ± 15.6 µg/mL and 1033.7 ± 17.9 µg/mL, respectively [6], while polar lipid extracts of *Nannochloropsis oceanica* have likewise been associated with antioxidant activity [73]; the contribution of the lipid fraction to radical scavenging therefore appears reproducible across species, although it depends markedly on lipid class and fatty-acid profile, which differ with strain, fraction and extraction protocol tested. Notably, certain fatty acids have been reported to act as important antibacterial agents, with broad-spectrum effects against Gram-positive bacteria such as *Staphylococcus aureus*, *B. cereus*, *S. pyogenes*, and *S. salivarius*. A study by [45] showed that a fraction enriched in palmitoleic acid exhibited increased antibacterial activity against *V. alginolyticus* and *V. parahaemolyticus* as the palmitoleic acid content rose, producing inhibition zone diameters of 10.37 ± 0.18 mm and 13.70 ± 0.38 mm, respectively, at 15 mg mL^−1^; it should be noted, however, that this activity was observed at a relatively high concentration, the pharmacological relevance of which remains to be established. In a separate study, lipid extracts from several microalgae were evaluated as photosensitizers in antimicrobial photodynamic therapy (aPDT), in which bactericidal activity is triggered by visible light irradiation rather than by the extracts alone [47]. Under these light-activated conditions, all extracts (at 1 mg mL^−1^) reduced *Staphylococcus aureus* by more than 3 log_10_ CFU mL^−1^, with the most effective extracts from *Phaeodactylum tricornutum* (Bacillariophyceae) and *Tisochrysis lutea* (Haptophyta) achieving reductions exceeding 6 log_10_ CFU mL^−1^, an effect attributed to their chlorophyll c, fucoxanthin, and polyunsaturated fatty acid content. By contrast, the *Limnospira platensis* (formerly *Arthrospira platensis*) extract was the least effective, achieving only a 4.4 log_10_ reduction, which illustrates the strong species-dependence of the activity. Taken together, these reports indicate antibacterial potential across several lipid-producing microalgae, but the activity is markedly dependent on the active compounds present, the tested concentration, the target organism, and, in the case of aPDT, on light activation. Moreover, these findings were obtained in vitro and the studies did not assess selectivity or cytotoxicity toward host cells; the results should therefore be regarded as preliminary indicators of antibacterial potential rather than evidence of therapeutic applicability, and their relevance to clinical antimicrobial resistance remains to be confirmed at physiologically achievable concentrations and with appropriate safety evaluation.

#### 4.2.4. Polysaccharides

In microalgae and Cyanobacteria, polysaccharides are found both as intracellular components and extracellular polymeric substances (EPS) [68]. In Cyanobacteria, polysaccharides are synthesized through central metabolic pathways such as glycolysis and the pentose phosphate pathway. In microalgae, polysaccharide production often includes the secretion of extracellular polymeric substances (EPS), a process that can be stimulated by interactions with associated microorganisms. These interactions involve the recognition of microbe-associated molecular patterns (MAMPs), which activate MAMP-dependent signaling pathways and regulate polysaccharide biosynthesis [101]. Cyanobacterial EPS are complex heteropolysaccharides of high molecular weight, often composed of six or more different monosaccharides. These polymers form protective layers around cells or cell clusters, playing key roles in biofilm formation, substrate adhesion, and resistance to biotic and abiotic stressors [42]. Their reported biological activities are closely tied to their structural and physicochemical characteristics, which differ considerably among the studies examined. For *Anabaena* sp. CCC 745, two distinct exopolysaccharide fractions—a capsular and a released form—were purified by dialysis and size-exclusion chromatography and shown to differ in molecular weight (30.3 and 19.6 kDa) and in uronic-acid content; notably, the released fraction, richer in glucuronic acid, exhibited the higher antioxidant and radical-scavenging activity [34]. The anti-inflammatory exopolysaccharide of *Chlorella vulgaris*, by contrast, is a high-molecular-weight (~845 kDa) proteoglycan with a complex, highly branched arabino-rhamno-galactan structure resolved by NMR, in which the associated protein fraction may itself contribute to the immunological response [23]; the same study notes that reported monosaccharide compositions for *C. vulgaris* exopolysaccharides vary markedly between research groups, a difference attributed to strain and cultivation conditions. A further degree of structural diversity is evident in the galactose-rich, heavily O-methylated proteoglycan of *Dictyosphaerium chlorelloides*, whose distinctive methylation pattern underlies a different activity profile altogether [69]. Anti-inflammatory activity has also been reported for exopolysaccharides from other sources: an exopolysaccharide from the marine dinoflagellate *Heterocapsa* sp. reduced IL-6 levels by about 46% and IL-8 secretion by up to 79% in stimulated cells [41], while exopolysaccharides from endolithic cyanobacteria, whose production increased under salinity stress, likewise showed significant anti-inflammatory potential [42]. This finding indicates that the physiological state of the producing strain and the environmental conditions under which it is grown, can itself potentially modulate both the amount and the bioactivity of the polysaccharides obtained. Taken together, these results show that anti-inflammatory activity extends across several polysaccharide-producing taxa, although the reported effects differ considerably in both magnitude and mechanism. Such variability is consistent with the strong dependence of polysaccharide bioactivity on monosaccharide composition, molecular weight, and degree of sulfation—features that are themselves shaped by the species, its physiological state, and the extraction method employed.

#### 4.2.5. Proteins

In this review, proteins were predominantly reported in studies addressing the nutritional and dietary applications of Cyanobacteria and microalgae, underscoring their relevance as high-quality and sustainable protein sources [88]. Cyanobacterial and microalgal proteins have gained global attention as food supplements and alternative protein sources, particularly from species like *Anabaena*, *Nostoc*, and *Limnospira*, which are known for their high protein and fiber content and are traditionally consumed as food. Notably, *Limnospira platensis* (formerly *Arthrospira platensis*) exhibits exceptionally high protein content, reaching up to 85.5% of dry weight [86], positioning it among the richest non-animal protein sources reported to date.

In microalgae, protein levels show greater interspecies variability but remain comparable to conventional animal and plant protein sources. For instance, *Dunaliella salina* and *Dunaliella viridis* display protein contents similar to chicken (24%), fish (24%), and peanuts (26%), with protein contents reported as 22.5 g/100 g DW and 19.67 g/100 g DW, respectively [96], while *D. salina* C13 and *Chlorella sorokiniana* exhibit markedly higher concentrations of 57 g/100 g DM and 62.5 g/100 g DM, respectively. These values, together with the variation observed even within a single genus, indicate that protein richness is a food-value characteristic shaped by species, strain, and cultivation conditions rather than a fixed property.

Distinct from their nutritional value, certain protein fractions also display functional bioactivity that may support pharmaceutical or cosmetic applications. For example, the same study reporting high protein content in *D. salina* C13 and *C. sorokiniana* also measured antioxidant activity (ABTS assay) in these strains [95], and phycobiliproteins have been associated with bioactive properties relevant to therapeutic and cosmetic formulations [72]. Such bioactivities, however, rest on a different and generally more limited body of evidence than the nutritional data and should be evaluated on their own terms rather than inferred from protein content alone.

Overall, despite their high protein content, challenges remain regarding digestibility, bioavailability, and large-scale processing, which are inconsistently addressed across studies. Future research should prioritize standardized analytical methods, evaluate protein functionality in vivo, and explore sustainable bioprocessing to fully establish both the nutritional and the functional potential of these organisms.

### 4.3. Antioxidant Potential

Oxidative stress, a hallmark of various chronic diseases, results from the imbalance between harmful reactive oxygen species (ROS) production and the body’s repair mechanisms. The analysis of the studies included in this review revealed a clear disproportion in the pharmacological applications investigated, with antioxidant activity being the most frequently assessed property compared to anti-inflammatory, antimicrobial, and other pharmacological effects. This highlights the strong research interest in the antioxidant potential of microalgae and Cyanobacteria extracts. Antioxidant capacity was predominantly evaluated using radical scavenging assays, particularly DPPH and ABTS [22,30,38]. In contrast, metal-reduction-based methods such as FRAP were less commonly applied [28]. DPPH results were generally expressed as IC_50_ values [27] or as percentage inhibition [33], depending on the study.

It is important to emphasize, however, that DPPH, ABTS, and FRAP are carried out in cell-free chemical systems and reflect mainly the intrinsic capacity of an extract to scavenge synthetic radicals or reduce metal ions. As such, the values they yield describe chemical behavior in solution and capture only part of what determines an antioxidant response in a living organism, where absorption, distribution, metabolism, and the cellular defenses against oxidative stress all come into play. A strong result in these tests therefore points to chemical reactivity rather than to a confirmed effect at the physiological level. Since the antioxidant evidence gathered here rests almost entirely on such assays, with few studies employing cellular or in vivo approaches, these findings are best read as an early indication of potential, awaiting confirmation in cellular, animal, and eventually human studies. Moreover, because the antioxidant and inhibitory values compiled in Table 1 were obtained with different solvents, assays, units (e.g., IC_50_ in µg/mL versus µmol TE/g), and test concentrations, they cannot be directly compared across studies; consequently, no genus, species, or compound can be designated as definitively “most effective” on this basis, and the comparative observations throughout this section should be read as qualitative indications of activity rather than as a validated ranking of potency.

Besides the diversity of methods used to evaluate antioxidant activity, the antioxidant composition of Cyanobacterial and microalgal extracts appears to be strongly influenced by several factors, particularly the extraction solvent and the extraction method employed [17], since the choice of solvent has been widely investigated in the literature because it directly affects the efficiency of bioactive compound recovery. For instance, ref. [30] reported that the methanolic extract of strain D1 exhibited the highest radical scavenging activity (36.7%) when tested at 0.5 mg/mL using the ABTS assay, compared to hexane (18.16%), chloroform (17.70%), and ethyl acetate (17.33%) extracts of the same strain. In contrast, ref. [27] demonstrated that only ethanol extracts from all tested species, as well as the aqueous extract of *Porphyridium* sp. (POC) and the ethyl acetate extracts of *Nannochloropsis* sp. (NANNO), *Phaeodactylum tricornutum* (PHA), and *Skeletonema* sp. (SKE), exhibited scavenging activities exceeding 50%. As solvent selection is closely related to the polarity of the target antioxidant compounds, variability in extraction methods and operational parameters can further influence antioxidant yields and bioactivity. Ref. [29] highlighted that extraction parameters such as time, pH, and temperature significantly affect the biological activity of *P. tricornutum* extracts. In their study, the highest antioxidant content (870 ± 22 µg TE/mL), as measured by the DPPH assay, was obtained under optimized conditions of 20 min extraction time, pH 7, and 33 °C. The authors further emphasized the importance of considering thermal degradation effects, noting that prolonged extraction durations and elevated temperatures can reduce antioxidant activity due to the degradation of thermolabile compounds.

### 4.4. Anti-Inflammatory Properties

Inflammation is a very complex biological process involving the secretion of mediators such as prostaglandins, leukotrienes, histamine, bradykinin, platelet-activating factor, and pro-inflammatory cytokines, including interleukin-1, produced by both resident tissues and migratory immune cells. Bioactive compounds derived from microalgae and Cyanobacteria can exert anti-inflammatory effects by modulating these inflammatory pathways. In this context, the regulation of key mediators such as cyclooxygenase-2 (COX-2), the downregulation of pro-inflammatory cytokines and cell-surface receptors, and the scavenging of reactive radicals play critical roles in controlling inflammatory responses [103]. The evaluation of anti-inflammatory potential in the included studies was largely shaped by the methodological approaches adopted. In vitro non-cellular assays, such as nitric oxide (·NO) scavenging [6] and cyclooxygenase-2 (COX-2) inhibition assays [40], were among the least explored. In contrast, in vitro cellular models were more commonly employed, with most studies using LPS or TNF-α [18,27]. For instance, available evidence indicates that the anti-inflammatory activity of Cyanobacterial and microalgal bioactive compounds is strongly dose-dependent. Ref. [27] reported that the TCTP4 ethanol extract induced a dose-dependent reduction in TNF-α production, achieving the highest inhibition (83.0 ± 9.4%) at 50 µg/mL, an effect significantly greater than that of the positive anti-inflammatory control dexamethasone (57.0 ± 8.6%; *p* < 0.001). Similarly, pretreatment with NANNO ET and POCW extracts resulted in significant TNF-α downregulation at concentrations of 50 µg/mL (*p* < 0.01) and 10 µg/mL (*p* < 0.001), as well as 50 µg/mL (*p* < 0.01), respectively. Consistent with these findings, ref. [41] demonstrated that IL-6 levels were reduced to 46.2% following treatment with exopolysaccharides (EPS) at concentrations above 500 µg mL^−1^ (*p* ≤ 0.01), while IL-8 secretion decreased by up to 79.3% across all tested concentrations. In contrast, ref. [18] reported that exposure of fibroblast cells to higher concentrations of phycoerythrin and phycocyanin pigments induced a significant pro-inflammatory response, and this increase in inflammation was significant up to a concentration of 1000 ppm (*p* < 0.05), highlighting a clear concentration-dependent dual effect. Despite these promising results, a critical gap remains. All three studies are preclinical and based on in vitro models; therefore, the observed dose-dependent anti-inflammatory effects must be interpreted cautiously until validated in vivo, where factors such as bioavailability, metabolism, systemic distribution, immune interactions, and potential toxicity play decisive roles. Collectively, these findings position Cyanobacteria- and microalgae-derived bioactive compounds as promising anti-inflammatory agents; however, their therapeutic potential ultimately depends on rigorous validation in whole-organism models and translational studies.

### 4.5. Antimicrobial Activity

Antimicrobial activity is one of the pharmacological potentials explored in this review. With antimicrobial resistance causing approximately 1.27 million deaths annually and contributing to a rising number of infections, antimicrobial activity has been identified by the Health Emergency Preparedness and Response Authority (HERA) as one of the top three global health threats [47]. Several microalgae and Cyanobacteria species have shown promising antimicrobial properties. For example, *Chlorella variabilis* extract inhibited cholera toxin production by 95% at 0.2% TLCV [53]. Additionally, a significant antimicrobial effect against *Staphylococcus aureus* was reported, with a MIC of 15.62 µg/mL and a MBC of 31.25 µg/mL [48]. Such activities have been attributed in another study to the presence of diverse bioactive metabolites, including proteins, polysaccharides, pigments, phenolic compounds, vitamins, and fatty acids, which collectively inhibit microbial growth and can interfere with bacterial quorum-sensing systems, thereby limiting colonization and infection [43]. In the same study, *Cladophora* extracts exhibited potent antibacterial activity against multiple pathogenic strains, with inhibition zones ranging from 17.7 to 18.3 mm. It is worth noting, however, that the inhibition of cholera toxin by *C. variabilis* [53] reflects an anti-virulence strategy that interferes with a pathogenic mechanism rather than killing bacteria, which differs mechanistically from conventional growth-inhibition screening and follows a distinct route toward application.

Beyond conventional extracts, recent studies have explored nanotechnology-based strategies, which constitute a separate category in which the organism serves as a platform or the active compound is reformulated, rather than acting directly as an antimicrobial extract. Ref. [49] reported that biogenic silver nanoparticles (AgNPs) synthesized using the cyanobacterium *Nostoc carneum* as a biological platform exhibited strong antibacterial effects against both Gram-negative and Gram-positive bacteria, primarily through the release of Ag^+^ ions that disrupt bacterial membranes, induce oxidative stress, and interfere with essential cellular processes. These effects were particularly pronounced in Gram-negative bacteria, likely due to differences in cell wall architecture that facilitate greater nanoparticle interaction and ion uptake. In contrast, ref. [50] demonstrated that microalgal oil-loaded nanoparticles produced a more moderate yet sustained antibacterial effect, attributed to enhanced nanoparticle stability and controlled release of bioactive compounds. These contrasting outcomes highlight a trade-off between immediate antimicrobial potency and controlled delivery: AgNPs induce rapid, largely concentration-independent bactericidal effects but raise concerns regarding cytotoxicity and environmental persistence, whereas nano-formulated microalgal oils emphasize stability and sustained release, features that may be more suitable for long-term or food-related uses. These approaches therefore carry safety and translational considerations distinct from those of crude extracts and should be weighed separately rather than grouped with conventional susceptibility data.

Taken together, these findings span different stages and strategies, from basic susceptibility screening to anti-virulence approaches and engineered nanomaterials, each with their own translational pathway and safety profiles. They are best regarded as complementary indications of antimicrobial potential rather than directly comparable evidence, and none, at present, constitutes demonstrated clinical anti-infective efficacy.

### 4.6. Other Pharmacological Potentials

Beyond the commonly studied antioxidant, anti-inflammatory, and antimicrobial activities, the included studies reveal a wide spectrum of other pharmacological applications associated with Cyanobacteria and microalgae. This category, though less frequently explored on an individual basis, collectively reflects a rich diversity of bioactivities with therapeutic promise. For instance, an in silico study employing a molecular docking approach demonstrated that microalgal strains such as *Limnospira platensis* (formerly *Spirulina platensis*) exhibit potential antidiabetic properties. Specifically, the bioactive compounds pheophytin, β-carotene, and phycocyanobilin showed higher binding affinities to α-amylase (by 0.4, 2.0, and 2.6 kcal/mol, respectively) than some commonly used commercial antidiabetic drugs, such as acarbose, suggesting a possible inhibitory effect on carbohydrate-digesting enzymes [55]. Because these results derive from a molecular docking approach (in silico), they represent computational predictions that require subsequent in vitro and in vivo validation before any antidiabetic effect can be inferred. Notably, *Haematococcus lacustris* (formerly *Haematococcus pluvialis*) extracts improved insulin sensitivity in vitro, in equine adipose-derived stem cells with palmitic-acid-induced insulin resistance [65], while *Dunaliella salina* carotenoid-rich fractions displayed marked benefits against obesity-related cardiac dysfunction in an in vivo rat model [20]. Other reported bioactivities included antiviral effects against SARS-CoV-2, with the included studies contributing complementary levels of evidence. Computational analyses based on virtual screening and molecular docking identified *Limnospira platensis* and *Microcystis aeruginosa* as promising sources of antiviral candidates [1,57], providing a valuable mechanistic rationale that warrants subsequent experimental confirmation. Consistent with this direction, *Nostoc edaphicum* exhibited direct antiviral activity in an in vitro assay (IC_50_ = 80 ng/mL) [63], which constitutes stronger experimental evidence. Other bioactivities also include neuroprotective and antidepressant activities (*Microcoleus autumnalis*—formerly *Phormidium autumnale*, *Botryococcus terribilis*) [64,66].

### 4.7. Nutritional and Dietary Applications

Among the various themes explored in the selected articles, nutritional and dietary applications emerged as the most frequently addressed aspects. Nutraceuticals are gaining significant popularity in the food industry due to their ability to serve as affordable nutritional sources while also offering therapeutic benefits and helping meet global nutritional demands [97]. This trend reflects the growing scientific interest in microalgae and Cyanobacteria as functional food ingredients and dietary supplements [96]. These organisms are rich in bioactive compounds such as carotenoids, polyphenols, and polysaccharides, which are believed to contribute to health-promoting effects, including the prevention of metabolic disorders [79]. Overall, this highlights the increasing recognition of microalgae and Cyanobacteria as sustainable and valuable nutritional resources for both human and animal consumption [73].

The increasing use of Cyanobacteria- and microalgae-based products in everyday human consumption is becoming more evident and is supported by both the European Food Safety Authority (EFSA) and the Food and Drug Administration (FDA). While numerous algae-based supplements are now commercially available and promoted for their health benefits, scientific evidence regarding their efficacy remains limited, and concerns about the safety of long-term and regular consumption are often overlooked. These supplements fall into a regulatory gray area—classified as foods yet exhibiting bioactivities potent enough to resemble medicinal products—raising concerns about their unrestricted use. The key challenge lies in the lack of clear regulations and international consensus on how to define, standardize, and ensure the quality and safety of these products [81].

A further consideration concerns the strength of the evidence underlying the reported bioactivities. The present review deliberately integrated in silico, in vitro, and in vivo studies to provide a comprehensive overview of the field; however, these models differ substantially in evidentiary value. The majority of the included studies were in vitro (72%), with a smaller proportion of in vivo (23%) and in silico (5%) investigations. Consequently, a large share of the reported activities—particularly antioxidant and enzyme-inhibition results derived from cell-free assays, and binding affinities derived from molecular docking—should be regarded as preliminary indicators of bioactivity rather than confirmed physiological effects. In silico predictions, in particular, require subsequent experimental validation, and in vitro findings do not necessarily translate to in vivo efficacy owing to differences in bioavailability, metabolism, and dosing. Hence, whenever possible, in vitro tests should be preferred over in silico tests for the evaluation of the effects of compounds derived from Cyanobacteria and microalgae, and there is also a need for further in vivo and, ultimately, clinical studies to substantiate the most promising findings.

## 5. Conclusions and Future Perspectives

In recent years, the global demand for healthcare and food has been rising due to population growth and an increasing awareness of sustainable and environmentally friendly natural resources. Cyanobacteria and microalgae, two groups of marine microorganisms, produce a wide variety of bioactive compounds such as carotenoids, polysaccharides, proteins, and lipids, which exhibit notable antioxidant, anti-inflammatory, and antimicrobial properties. In addition to their therapeutic potential, these organisms are also increasingly explored for their nutritional and dietary applications, notably as rich sources of essential nutrients that may contribute to weight management, metabolic health, and overall well-being. The studies included in this review emphasize the biotechnological potential of Cyanobacterial and microalgal extracts or biomass as sustainable, natural sources of phytochemicals for use in pharmaceutical and nutritional applications. However, despite their extensive biodiversity, research has disproportionately focused on a limited number of genera, such as *Limnospira platensis* (formerly *Arthrospira*/*Spirulina*), *Chlorella vulgaris*, and *Nannochloropsis* spp. This narrow scope may overlook the investigation of numerous other species with untapped biotechnological potential, particularly in pharmaceutical and nutraceutical applications. Expanding research efforts to screen a broader range of taxa could therefore unveil new sources of valuable bioactive compounds. A further gap concerns the level of chemical resolution: most studies assessed crude extracts (68.96%) or broad fractions (pigments, lipids, proteins, polysaccharides), with very few isolating and purifying individual compounds to attribute activity to a defined molecule. Future work should therefore prioritize bioassay-guided isolation and the structural and functional characterization of purified compounds. Nevertheless, it is important not to overlook the limitations associated with the use of bioactive compounds derived from Cyanobacteria and microalgae, which were less thoroughly addressed in many of the studies included in this review. These limitations primarily relate to high production costs, as obtaining high-purity and standardized extracts of valuable bioactive compounds (e.g., phycocyanin, astaxanthin) remains technically challenging and economically demanding. In addition, the instability of certain compounds, along with high operational and maintenance costs and difficulties associated with large-scale cultivation and downstream processing, poses significant barriers to industrial-scale applications. Furthermore, safety and toxicity concerns were insufficiently discussed across the reviewed studies, as the emphasis was predominantly placed on the beneficial biological effects of bioactive compounds derived from different strains rather than on the potential toxicity of the producing organisms themselves or on the safety of their use in humans. This review presents an original and updated overview demonstrating that Cyanobacteria- and microalgae-based products hold significant potential as antioxidant, anti-inflammatory, and antimicrobial agents, along with other pharmacological properties such as anticancer, antiviral, and cardioprotective effects, as well as nutritional and dietary roles. Nevertheless, it must be emphasized that this evidence derives from in vitro and, in some cases, in silico investigations. While in silico approaches help predict and prioritize promising candidates, and in vitro assays demonstrate activity under controlled conditions, neither can establish the safety or efficacy of these compounds in living systems. Confirming these bioactivities therefore requires progression toward in vivo models and, ultimately, clinical evaluation, supported by rigorous toxicological and regulatory-oriented studies to enable the safe and sustainable translation of Cyanobacterial and microalgal bioactivities into nutraceutical and pharmaceutical applications. Within this validated framework, their multifunctionality stands as a major strength, underscoring the need for greater investment from the pharmaceutical industry to shift toward sustainable, safe, and natural compound sources.

## Figures and Tables

**Table 1:** Summary of the main findings on the pharmacological properties of extracts and biomass derived from Cyanobacteria and microalgae.

Phylum	Family	Species	Type of Extract and Method of Extraction	Activity	Main Finding	Possible Pharmaceutical/Therapeutic Application	References
Cyanobacteria	Microcystaceae	*Gloeothece* sp.	Polar lipids: Solvent-based (Methanol/Chloroform, Biphasic Extraction)	Antioxidant/anti-inflammatory	IC_50_ (μg mL^−1^): (O_2_·^−^): 939.8 ± 15.6 (·NO): 1033.7 ± 17.9. (COX-2): 58% inhibition of PG2.	NR	[6]
Cyanobacteria	Nodulariaceae, Prochlorococcaceae, Oscillatoriaceae, Microcoleaceae, Spirulinaceae, Nostocaceae, Nostocaceae, Nostocaceae, Desertifilaceae	*Nodularia* sp. (CCM-UdeC 012), *Cyanobium* sp. (CCM-UdeC 013), *Phormidium* sp. (CCM-UdeC 014), *Nodularia* sp. (CCM-UdeC 015), *Limnospira maxima* (formerly *Arthrospira maxima*) (CCM-UdeC 040), *Spirulina subsalsa* (CCM-UdeC 050), *Nostoc* sp. (CCM-UdeC 087), *Nostoc linckia* (CCM-UdeC 088), *Anabaena iyengarii* (CCM-UdeC 089), *Desertifilum* sp. (CCM-UdeC 135).	Crude extract or biomass: Aqueous extraction (water-based)—cold maceration (stirring at room temperature for 24 h)	Antioxidant/anti-inflammatory	DPPH: CCM-UdeC 012 and CCM-UdeC 015 strains (8.12 and 8.83 μmol TE g^−1^). ABTS and FRAP: CCM-UdeC 050 strain (33.26 ± 1.71, 40.97 ± 4.35 μmol TE/g^−1^) and CCM-UdeC 135 strain (33.69 ± 2.36, 36.93 ± 4.95 μmol TE/g^−1^). Percentage of hemolysis: CCM-UdeC 135 (56.7%)-CCM-UdeC 014 (52.8%)	NR	[26]
Chlorophyta, Rhodophyta, Heterokontophyta, Haptophyta, Heterokontophyta, Heterokontophyta, Cyanobacteria, Chlorophyta, Chlorophyta	Chlorodendraceae, Porphyridiaceae, Monodopsidaceae, Isochrysidaceae, Phaeodactylaceae, Skeletonemataceae, Spirulinaceae, Haematococcaceae, Chlorodendraceae	*Tetraselmis striata* CTP4, *Porphyridium* sp., *Nannochloropsis* sp., *Tisochrysis lutea*, *Phaeodactylum tricornutum*, *Skeletonema* sp., *Limnospira* (formerly *Spirulina)*, *Haematococcus lacustris* (formerly *Haematococcus pluvialis*), *Tetraselmis chui*	*Crude* extract or biomass: solvent extraction (Ethanol; ethyl acetate; water)	Antioxidant/anti-inflammatory	*P. tricornutum* (ethyl acetate extract) IC_50_: 0.452 ± 0.002 mg/mL *Skeletonema* sp. (ethyl acetate extract) IC_50_: 0.447 ± 0.060 mg/mL *H. pluvialis* (ethanol extract) IC_50_: 0.408 ± 0.007 mg/mL. TNF-α Inhibition: significant downregulation of TNF-α production (*p* < 0.001)	Anticancer	[27]
Rhodophyta	Porphyridiaceae	*Porphyridium purpureum*	Crude extract or biomass: cold plasma-assisted extraction	Antioxidant	DPPH: (69.44 ± 0.10%). FRAP: 207.29 ± 12.96 μmol Fe^2+^/g	Anticancer	[28]
Bacillariophyta	Phaeodactylaceae	*Phaeodactylum tricornutum*	Crude extract or biomass: Infrared-assisted extraction, Water-based solvent	Antioxidant/Antimicrobial	DPPH: 870 µg TE/mL. *S. aureus* inhibition zones (~2 mm)	Antimicrobial	[29]
Cyanobacteria	Nostocaceae	*Desmonostoc alborzicum*	Pigments: cell disruption, Freeze–thaw extraction	Antioxidant/Antimicrobial	DPPH: 4.02 ± 0.10. Inhibition zone against: *Citrobacter freundii* (7.00 ± 0.00) *Escherichia coli* k12 (4.16 ± 1.44). Antifungal effect against *Saprolegnia parasitica* (6.66 ± 0.47).	Antimicrobial	[22]
Chlorophyta	Chlorellaceae	*Chlorella* sp.	Crude extract or biomass: chloroform, methanol, ethyl acetate and ethanol.	Antioxidant	ABTS (methanol extract): 36.7% at 0.5 mg/mL DPPH (methanol extract): 34.10% at 1 mg/mL FRAP (methanol extract): 237.08 μM Fe^2+^ at 1 mg/mL.	Anticancer/neuroprotectivity	[30]
Cyanobacteria	Spirulinaceae	*Limnospira platensis* (formerly *Spirulina platensis*) PCC 7345	Pigments: phycocyanin, freeze–thaw method with sodium phosphate buffer (1 M, pH 7.0)	Antioxidant	DPPH- IC_50_: 40.70 µg/mL ABTS- IC_50_: 23.25 µg/mL NO- IC_50_: 17.74 µg/mL	Antidiabetic	[31]
Cyanobacteria	Tolypothrichaceae, Nostocaceae, Scytonemataceae, Oculatellaceae	*Tolypothrix* sp. LEGE 221228, *Nostoc* sp. LEGE 221229, *Scytonema* sp. LEGE 221230, *Drouetiella* sp. LEGE 221231	Crude extract or biomass: organic solvent-based extraction with acetone—Aqueous extraction (water-based solubilization)	Antioxidant	(O_2_·^−^): *Tolypothrix* sp. LEGE 221,228 IC_50_ (53.75 μg/mL). (·NO): *Tolypothrix* sp. LEGE 221,228 IC_50_ (1220.00 ± 39.60).	NR	[32]
Heterokontophyta, Rhodophyta	Phaeodactylaceae, Monodopsidaceae, Porphyridiaceae	*Phaeodactylum tricornutum*, *Nannochloropsis oculata*, and *Porphyridium purpureum* (formerly *Porphyridium cruentum*)	Lipids: supercritical CO_2_ extraction (SC-CO_2_)/subcritical n-Butane extraction	Antioxidant	DPPH: (44.6%)	NR	[33]
Chlorophyta	Chlamydomonadaceae	*Chlamydomonas agloeformis*	Crude extract or biomass: solvent extraction using 80% methanol/water solution, cold maceration/solid–liquid extraction.	Antioxidant	ORAC (µmolTE/100 g DW): 4827.66 ± 1.33 FRAP (µmolTE/100 g DW): 45.53 ± 5.53 DPPH (µmolTE/100 g DW): 375.51 ± 3.6	NR	[17]
Cyanobacteria	Aphanizomenonaceae	*Anabaena* sp. CCC 745	Polysaccharides: Salt/EDTA-assisted extraction + dialysis + precipitation (for EPS purification)	Antioxidant	RPS scavenging activity: 71.77%	NR	[34]
Chlorophyta	Chlorellaceae	*Micractinium* sp. ME05	Crude extract or biomass: Ultrasound-assisted solvent extraction.	Antioxidant	DPPH: 7.72 ± 0.95%/93.80 ± 6.28 µmol TE/g DW	Cytoprotective	[35]
Cyanobacteria	Leptolyngbyaceae	*Leptolyngbya* sp. KC45	Crude extract or biomass: microwave-assisted solvent extraction using ethanol	Antioxidant	DPPH: IC_50_ = 35.51 mg/mL ABTS: IC_50_ = 3.31 mg/mL PFRAP: 12.51 mg GAE/g	Anticancer—enzyme related disease inhibition	[36]
Chlorophyta	Haematococcaceae	*Haematococcus* sp. CCAP 34/7, LAFW15, RPFW01	Pigment (astaxanthin): 100% acetone extract, saponification using methanolic NaOH	Antioxidant	Strain RPFW01: highest FC activity (62.9–155.0 µmoL g^−1^ of DW)	NR	[37]
Heterokontophyta	Monodopsidaceae	*Nannochloropsis oceanica*	Lipids: Solvent Extraction: Chloroform/methanol (2:1, *v*/*v*) Folch method-based, Dichloromethane/methanol (2:1, *v*/*v*), Dichloromethane/ethanol (2:1, *v*/*v*)—Lipid Purification.	Antioxidant	ABTS (all extracts): 90% of inhibition under 500 µg/mL DPPH (E + USP extract): 55–60% of inhibition under 500 µg/mL	NR	[38]
Cyanobacteria	Nostocaceae	*Nostoc* NIES 2111_MUM004	Crude extract or biomass: ultrasound-assisted aqueous extraction	Antioxidant	ABTS: 10.65 ± 0.34 mg FRAP: 110.71 mg β-carotene bleaching assay: 537.98 ± 58.15 mg	wound healing properties	[39]
Cyanobacteria	Nostocaceae, Spirulinaceae	*Nostoc commune*, *Limnospira platensis* (formerly *Spirulina platensis*)	Pigments: freeze–thaw aqueous buffer extraction	Anti-inflammatory	PE and PC extracts at: 1.95 ppm and 62.5 ppm for IL-6 release 7.81 ppm and 31.25 ppm for TNF-α release	Anticancer	[18]
Chlorophyta	Chlorellaceae	*Chlorella vulgaris* (BEIJ. 1890, strain P13/1998)	Polysaccharides/Method of extraction: NR	Anti-inflammatory	EPS50 elevated IL-12 levels: 13 pg.mL^−1^, *p* ≤ 0.05. IFN-γ levels were significantly increased in the EPS50 group: 33 pg.mL^−1^.	Anti-remodelling, anti-asthmatic	[23]
Cyanobacteria	Oscillatoriaceae	*Lyngbya* sp.	Crude extract or biomass: mechanical Lysis/cryogenic grinding with Liquid nitrogen, solvent extraction (Methanol/Chloroform 1:2), (Hexane:Ethanol:Methanol 2:1:1).	Anti-inflammatory/antioxidant	COX-2 Inhibition: IC_50_ = 117.98 ± 0.41 µg/mL DPPH: IC_50_ = 25.89 ± 0.21 µg/mL	NR	[40]
Cyanobacteria	Spirulinaceae	*Limnospira platensis* (formerly *Spirulina platensis*)	Pigments: C-phycocyanin = buffer-based mechanical disruption, freeze–thaw cycles	Anti-inflammatory/antioxidant/antiviral/anti-tumour	Inflammatory activity: 98.76 ± 0.065% DPPH: 99.12 ± 0.027%. Reduced viral infectivity by 56%, SI up to 36.76%. Anticancer activity: inhibiting up to 70% of liver (HepG-2), 74% of colon (HCT-116) and 62% of breast (MCF-7) cancer.	Anticancer	[21]
Dinoflagellata	Heterocapsaceae	*Heterocapsa* strain AC210	Polysaccharides: dialysis-based purification of EPS, Initial separation of the culture broth at 11,000× *g* for 45 min.	Anti-inflammatory/antioxidant	IL-8 Reduction: 79.3%. IL-6 Suppression: 46.2%. ROS inhibition: 18.3% at the highest concentration.	NR	[41]
Chlorophyta	Chlorellaceae	*Auxenochlorella pyrenoidosa* (formerly *Chlorella pyrenoidosa*)	Crude extract or biomass: Ultrasound-assisted extraction = 0.5 g of processed CP → 50 mL of ultrapure water → Ultrasonication, 250 W/40 kHz/30 min	Anti-inflammatory	Reduced inflammatory cytokines (IL-6, IL-1β, TNF-α) Increased protective markers (SIgA, IL-22). Reduced pro-inflammatory cytokines (IFN-γ, IL-17, IL-23).	Ulcerative colitis therapy	[3]
Cyanobacteria	Gloeocapsaceae, Chroococcidiopsidaceae, Gloeocapsaceae, Chroococcidiopsidaceae, Acaryochloridaceae	*Gloeocapsa* sp. UAM572, *Chroococcidiopsis* sp. UAM574, *Gloeocapsopsis* sp. UAM575, *Chroococcidiopsis* sp. UAM577, *Chroococcidiopsis* sp. UAM579, *Chroococcidiopsis* sp. UAM584, *Pseudoacaryochloris* sp. UAM587.	Polysaccharides: The dry biomass was resuspended in 1 mL of Milli-Q water → EPS quantification.	Anti-inflammatory	Best inhibitor against neutrophil elastase: *Chroococcidiopsis* sp. UAM579—IC_50_ = 78 μg EPS/mL.	Antielastase	[42]
Chlorophyta	Cladophoraceae, Chlamydomonadaceae	*Cladophora*, *Chlamydomonas*	Crude extract or biomass: hot aqueous extraction method	Antimicrobial	Inhibition zones: 17.7 mm to 18.3 mm. MIC: 10.0 MBC: 12.0 mg/mL in the case of *S. aureus* bacteria.	NR	[43]
Chlorophyta	Chlorellaceae, Scenedesmaceae	*Chlorella vulgaris*, *Scenedesmus incrassatulus*	Crude extract or biomass: sonication-assisted microwave extraction method	Antimicrobial/antioxidant	MICs: 16 mg/mL against *P. aeruginosa* VOC extracts MICs: 1–2 mg/mL DPPH: SIDES2 (*S. incrassatulus*, IC_50_ = 3.98 mg/g) and CVDES1 (*C. vulgaris*, IC_50_ = 5.58 mg/g).	NR	[44]
Heterokontophyta	Eustigmataceae	*Eustigmatos* cf. *Vischeria polyphem* (formerly *Eustigmatos polyphem*)	Lipids: Freeze-dried biomass was suspended in 95% ethanol, ultrasound treatment at 40 °C for 60 min.	Antimicrobial/antioxidant	ORAOs: inhibition zones of 9.21 ± 0.51 mm and 9.68 ± 0.38 mm, against *V. alginolyticus* and *V. parahaemolyticus*, respectively. ABTS: ORAOs achieved 68.27% scavenging activity	NR	[45]
Cyanobacteria	Spirulinaceae	*Limnospira platensis* (formerly *Spirulina platensis*) PCC 7345	Pigments: 20 g of phycocyanobilin-based colorant Linablue G1 suspended in 250 mL of 96% ethanol. Heated at 95 °C in an oil bath. Cooled, filtered through a Büchner funnel.	Antimicrobial	MICs > 64 μg mL^−1^	NR	[46]
Cyanobacteria, Chlorophyta, Heterokontophyta	Microcoleaceae, Chlorococcaceae, Monodopsidaceae, Phaeodactylaceae, Scenedesmaceae, Chlorodendraceae	*Limnospira platensis* (formerly *Arthrospira platensis*), *Oophila amblystomatis* (formerly *Chlorococcum amblystomatis*), *Nannochloropsis oceanica*, *Phaeodactylum tricornutum*, *Tetradesmus obliquus* (formerly *Scenedesmus obliquus*), *Tetraselmis chui*	Lipids: Folch extraction method: Biomass (25 mg) was homogenized with a 2:1 (*v*/*v*) dichloromethane:methanol solution, drying and re-extraction, Centrifugation at 2000 rpm/10 min.	Antimicrobial	Lipid extracts from *P. tricornutum*, *S. costatum*, *T. lutea*, and *P. gyrans*: ≥6 log_10_ CFU mL^−1^ bacteria reduction. Residual activity was noted for *P. gyrans*, *T. lutea*, *S. costatum*, and *D. salina*, causing reductions of 0.6–3.6 log_10_ CFU mL^−1^	NR	[47]
Heterokontophyta	Monodopsidaceae	*Nannochloropsis oculata*	Crude extract or biomass: ultrasound-assisted extraction with methanol	Antimicrobial/antioxidant	Strongest inhibition against *S. aureus*: MIC = 15.62 µg/mL, MBC = 31.25 µg/mL. DPPH assay: IC_50_ = 52.10 ± 0.85 µg/mL H_2_O_2_ assay: IC_50_ = 122.84 ± 2.32 µg/mL ABTS assay: IC_50_ = 96.95 ± 1.23 µg/mL	Anticancer	[48]
Cyanobacteria	Nostocaceae	*Nostoc carneum*	Crude extract or biomass Method of extraction: NR	Antimicrobial	Inhibition zone: 15 mm at the lowest concentration of AgNPs (5 µg) against *Pseudomonas aeruginosa*	Anticoagulative	[49]
Chlorophyta, Cyanobacteria	Botryococcaceae, Microcystaceae	*Botryococcus braunii*, *Microcystis aeruginosa*	Crude extract or biomass: Soxhlet extraction with ethanol	Antimicrobial	Inhibition zones: 20.00 ± 0.30 mm (BB oil) and 25.00 ± 0.20 mm (MA oil) against *E. coli* ATCC 8739.	NR	[50]
Heterokontophyta, Heterokontophyta, Chlorophyta, Chlorophyta, Chlorophyta, Cyanobacteria	Bacillariaceae, Staurosiraceae, Chlorellales, Chlorodendraceae, Chlorodendraceae, Halothecacae	*Nitzschia* sp. S5, *Nanofrustulum shiloi* D1, *Picochlorum* sp. D3, *Tetraselmis* sp. Z3, *Tetraselmis* sp. C6, *Euhalothece* sp. C1	Crude extract or biomass: Ultrasound-assisted extraction using 100% methanol, 25–50 °C, 1 h.	Antimicrobial/antioxidant	*Tetraselmis* sp. C6 showed the strongest antibacterial activity against *E. coli*: 26.05 ± 0.07 mm TEAC > 40 µmol TE/g *Tetraselmis* sp. C6 had strong free radical scavenging capacity (0.87 ± 0.23 mg/mL).	NR	[51]
Cyanobacteria	Oscillatoriaceae	*Phormidium papyraceum* PACC 8600	Crude extract or biomass: Solvent extraction with ultrasound-assisted extraction.	Antimicrobial	20.28 mm inhibition zone against *E. coli*.	Immunomodulatory	[52]
Chlorophyta	Chlorellaceae	*Chlorella variabilis* (ATCC PTA 12198)	Lipids: Ultrasonication-assisted solvent extraction (Chloroform:Methanol—2:1)	Antimicrobial	Inhibition of Cholera Toxin (CT) Production: highest inhibition (95%) was observed with 0.2% TLCV.	Anti-virulence	[53]
Cyanobacteria, Heterokontophyta	Spirulinaceae, Catenulaceae	*Limnospira platensis* (formerly *Spirulina platensis*), *Amphora coffeaeformis*	Crude extract or biomass: aqueous extraction assisted by sonication, followed by centrifugation and lyophilization	Antitoxicity/antioxidant	Extracts markedly mitigated AFB_1_ induced toxic effects. Red blood cells 10^6^/mm^3^: 6.02 ± 0.11 DPPH Assay: *Limnospira* extract: 179.56 ± 3.05 μmol TE/g DW *Amphora* extract: 141.66 ± 2.51 μmol TE/g DW ABTS Assay: *Limnospira* extract: 194.18 ± 3.21 μmol TE/g DW *Amphora* extract: 136.52 ± 1.14 μmol TE/g DW	NR	[54]
Cyanobacteria	Spirulinaceae	*Limnospira platensis* (formerly *Spirulina platensis*)	NR	Antidiabetic type-2	α-Amylase Binding affinity: Pheophytin 0.4 kcal/mol Phycocyanobilin 2.6 kcal/mol β-carotene 2.0 kcal/mol Zeaxanthin 1.4 kcal/mol α-Glucosidase: Pheophytin 2.1 kcal/mol.	NR	[55]
Chlorophyta	Scenedesmaceae	*Coelastrella* sp. SDEC-28	Crude extract or biomass: ultrasound-assisted solvent extraction using methanol, 1:20 (*m*/*v*) for crude polysaccharides, chloroform/methanol (2:1, *v*/*v*) mixture for lipids.	Anti-tumour/antioxidant	Methanol Extract: 76.61% inhibition of A375 cell at 1000 μg/mL IC_50_ of ME before purification: 357.81 µg/mL IC_50_ of ME after purification: 73.69 µg/mL DPPH· scavenging (crude polysaccharides): 91.18% at 2 mg/mL ABTS· scavenging (crude polysaccharides): 96.17% at 2 mg/mL	Anticancer	[56]
Cyanobacteria	Microcystaceae, Nostocaceae, Microcoleaceae	*Microcystis aeruginosa* NIES-298/PCC 9807, *Nostoc calcicola*, *Nostoc* sp., *Planktothrix rubescens*	NR	Antiviral	*Nostoc calcicola* with high carbohydrate affinity and favorable binding dynamics. Binding Free Energies: Site A: (Mannose Binding): 10–28 kcal/mol Site B: 2–26 kcal/mol	NR	[57]
Cyanobacteria, chlorophyta	Microcoleaceae, Chlorellaceae, Dunaliellaceae, Haematococcaceae	*Limnospira maxima* (formerly *Arthrospira maxima*) (LEAF046), *Chlorella vulgaris* (LEAF749), *Dunaliella salina* (LEAF754), *Haematococcus lacusrtis* (formerly *Haematococcus pluvialis*)	Crude extract or biomass: solvent extraction using DMSO	Antiviral	Viral Inhibition (IC_50_ values) against MAYV: *H. pluvialis*: 17.53 µg/mL *D. salina*: 16.00 µg/mL *C. vulgaris*: 12.19 µg/mL *A. maxima*: 36.47 µg/m	NR	[58]
Chlorophyta	Chlorellaceae	*Mychonastes homosphaera* (formerly *Chlorella minutissima*)	Crude extract or biomass: Soxhlet extraction with sequential solvent polarity (Hexane, Chloroform, Ethyl acetate, Acetone, Methanol, Water)	Antidiabetic type-2	*C. minutissima* acetone extract exhibited α-Glucosidase inhibition potential with an IC_50_ value of 1.61 mg·mL^−1^.	NR	[59]
Chlorophyta	Chlorellaceae	*Chlorella* sp. AT1	Pigments: Two-stage solvent extraction (Methanol + Diethyl ether/60% KOH).	Antidiabetic type-2	Reduced glucose intolerance by 25% (*p* < 0.01) Reduced insulin resistance by 41% compared to diabetic mice (*p* < 0.05).	Antidiabetic	[19]
Heterokontophyta	Chaetocerotaceae	*Chaetoceros neogracilis* (formerly *Chaetoceros gracilis*)	Crude extract or biomass: solvent extraction followed by sonication-assisted extraction/methanol:chloroform:water mixture in a ratio of 2:1:2	Anticancer/antidegenerative/anti-inflammatory/cardioprotective	Cancer-related pathways: 130 significant metabolic pathways (*p* ≤ 0.05) and gene counts >20. Neuroactive ligand-receptor interaction: 37 gene targets. Cardioprotective pathways: ligands binding with MTOR ranged from 6.981 to 5.149 kcal/mol.	NR	[4]
Cyanobacteria	Spirulinaceae	*Limnospira platensis* (formerly *Spirulina platensis*)	Crude extract or biomass: Ultrasound-assisted hydroalcoholic extraction (70% ethanol, 35 °C + 2 h).	Anti-rheumatoid arthritis/anti-inflammatory	SP significantly reduced: IL-6 by 46% MCP-1 by 46% MMP-9 by 41% TNF-α by 33% IL-1β by 49% IL-17 by 37% TLR4 (26%), NLRP3 (34%), ASC (37%) Pro-caspase-1 (32%), Caspase-1 (28%), NF-κB p65 (32%)	Anti-inflammatory reactions of RA	[60]
Chlorophyta	Scenedesmaceae	*Pectinodesmus* sp. PHM3	Crude extract or biomass: Hydrothermal carbonization	Anticancer	On HCC 1954 (breast cancer) cells: CA nanodots: GI_50_ = 0.316–0.447 ng/mL CG nanodots: GI_50_ = 8.156–6.596 ng/mL On HCT 116 (colorectal cancer) cells: CA nanodots: GI_50_ = 0.542–0.715 ng/mL CG nanodots: GI_50_ = 23.860–14.524 ng/mL	Anticancer	[61]
Heterokontophyta	Skeletonemataceae	*Skeletonema marinoi*	Crude extract or biomass: Ultrasound-assisted extraction with methanol	Anticancer	100% blockage of cells in the sub-G1 phase of the cell cycle after 48 h at 500 µg/mL	Anticancer	[62]
Cyanobacteria	Microcoleaceae	*Limnospira platensis* (formerly *Arthrospira platensis*)	Pigments: Maceration extraction assisted by vortexing and centrifugation	Antiviral	C-PC binds strongly to the ACE2: 9.7 kcal/mol. C-PC binds to ACE2 with high affinity: KD = 3.37 nM.	Anti-SARS-CoV-2	[1]
Cyanobacteria	Nostocaceae	*Nostoc * *edaphicum* CCNP1411	Crude extract or biomass: 150 g of biomass was homogenised by grinding and extracted five times with 75% methanol → vortexing (15 min) → sonication in ultrasonic bath (10 min) → centrifugation	Antiviral	Strongest antiviral activity by CCNP1411, IC_50_ = 80 ng/mL	Anti-SARS-CoV	[63]
Chlorophyta, Heterokontophyta, Chlorophyta, Chlorophyta, Heterokontophyta	Chlorellaceae, Monodopsidaceae, Chlorodendraceae, Chlorococcaceae, Phaeodactylaceae	*Chlorella vulgaris*, *Nannochloropsis oceanica*, *Tetraselmis * *chui*, *Oophila amblystomatis* (formerly *Chlorococcum amblystomatis*), *Phaeodactylum tricornutum*	Crude extract or biomass: High-pressure extraction = 25 mg of dried microalgae → Water, ethanol (48% or 96%), or acetone	Anticytotoxic	Low cytotoxicity (IC_50_ > 500 μg/mL) except for *P. tricornutum* in fibroblasts (IC_50_ < 150 μg/mL). Higher sensitivity (IC_50_ < 500 μg/mL) was observed in keratinocytes and melanocytes for most extracts.	Cosmetics	[12]
Chlorophyta	Botryococcaceae	*Botryococcus terribilis*	Crude extract or biomass: cold maceration extraction = 70% ethanol, room temperature, dark, 2 weeks	Antidepressant-like	Significant decrease in immobility time from day 2 onward (65.5 ± 6.1 s, 62.0 ± 9.8 s, 56.8 ± 13.3 s, 68.8 ± 8.3 s, 73.5 ± 4.6 s, 70.3 ± 11.9 s, and 71.7 ± 8.4 s on days 1–7, respectively) (*p* < 0.01). Significantly decreased (*p* < 0.01) expressions of BCL2, CD14, CD40LG, CXCL17, ICAM1, LY96, PLAU, and TRAF3 (74.1 ± 1.1%, 63.6 ± 1.6%, 62.7 ± 9.6%, 55.1 ± 4.0%, 51.7 ± 4.3%, 76.7 ± 5.8%, 60.4 ± 1.0%, and 63.0 ± 3.2%, respectively).	Anti-neurodegenerative	[64]
Chlorophyta	Haematococcaceae	*Haematococcus lacustris* (formerly *Haematococcus pluvialis*)	Crude extract or biomass: Ultrasound-assisted extraction with solvents (water and methanol)	Insulin resistance	Significant enhancement of EqASCs metabolic activity: 10%, 15%, and 20% (*v*/*v*) of the water extract. Decreased expression of mRNA for (IRS1): 0.3–0.4 Ct [target gene/GAPDH].	Insulin sensitivity	[65]
Cyanobacteria	Oscillatoriaceae	*Microcoleus autumnalis* (formerly *Phormidium autumnale*)	Crude extract or biomass: T1 = Solvent extraction with heptane for 24 h. T2 = Chemical saponification with KOH (1 mL; 6% *w*/*v*) + chloroform (0.5 mL) and hexane (4 mL).	Neuroprotective	Acetylcholinesterase inhibition: IC_50_ = 65.80 μg/mL Lipoxygenase inhibition: IC_50_ = 58.20 μg/mL Antioxidant activity: IC_50_ = 7.40 μg/mL	Anticholinergic	[66]
Chlorophyta	Chlorellaceae, Monodopsidaceae, Chlorodendraceae, Chlorococcaceae, Phaeodactylaceae	*Chlorella* sp. HS5	Crude extract or biomass: ethanolic extraction using high-pressure stirred extraction = Lyophilized *Chlorella* (400 kg) → 95% ethanol (1:10 biomass-to-solvent)	Retinoprotective	Reduced formation of oxidized A2E: IC_50_ = 30.28 ± 4.77 µg/ML. CEE restored cell viability dose-dependently by 98%. DCFH assay: Reduced oxidative stress. TUNEL assay: Decreased apoptosis. ONL thickness dropped after light exposure (from ~51 µm to ~16 µm).	Retinal degeneration	[67]
Chlorophyta	Chlorellaceae	*Brachionococcus chlorelloides* (formerly *Dictyosphaerium chlorelloides*)	Polysaccharides: algal biomass was removed by centrifugation at 2260× *g* → 96% ethanol precipitation in a ratio of 1:4 (*v*/*v*) → centrifugation at 3230× *g* → Freeze drying	Antitussive	The Dch biopolymer at a dose of 50 mg/kg significantly suppressed cough reflex to 2/300 min compared to control (8/300 min).	Antitussive	[68]
Cyanobacteria	Aphanizomenonaceae	*Aphanizomenon flos-aquae*	NR	Antispasmodic	Klamin (5–35 mg/mL) caused a dose-dependent decrease in the amplitude of the contractions (up to ~52% at 30 mg/mL).	Spasmolytic	[69]
Chlorophyta	Dunaliellaceae	*Dunaliella salina*	Crude extract or biomass: Solvent extraction by maceration (solvent: ethyl acetate:n-hexane 20:80, *v*/*v*)	Antiobesity	Improved adiponectin: +20.26%. Glucagon decreased: −41.43% in obese rats. Improved troponin I and PAI-1 levels: 46.06% and 52.15%. ICAM: +163.29%, VCAM: +35.38%, CRP: +84.48%. Col II: +112.52%, Col 3A1: +127.83%, LOX: +98.00% crf-DS amelioration: Col II: −83.30%/Col 3A1: −103.42%/LOX: −76.00%	Obesity-associated cardiac dysfunction	[20]
Cyanobacteria	Microcoleaceae	*Limnospira platensis* (formerly *Arthrospira platensis*)	NR	Antioxidative stress-mediated liver damage	liver/body weight ratio reduced to 121.2% SP/LA group: Blood lead decreased to +187.5% Liver lead decreased to +2666.6% TAC increased by 68% SOD & GSH improved CAT returned to normal MDA decreased to 256%	Antioxidative stress caused by metal exposure	[70]

NR: Not reported or not specified in the original study.

**Table 2:** Summary of the main findings on the biochemical composition and nutraceutical properties of Cyanobacteria- and microalgae-derived extracts and biomass.

Phylum	Family	Species	Method of Extraction	Biochemical Composition	Application	Main Findings	References
Heterokontophyta	Phaeodactylaceae	*Phaeodactylum tricornutum*	Heat-assisted extraction = Water/ethanol mixture (75/25, *v*/*v*) → 40 °C/80 °C, 1 h. Biomass concentration: 2% dried *P. tricornutum* in 100 mL solvent	42% of crude protein, 18% carbohydrates, 12% lipids, and 33% of ash. Monosaccharides = Glucose (E40: 6.3 ± 0.8%)/Man, Gal, Xyl (E80: 4.3 ± 0.2%)/Fucose (E80: 2.9 ± 0.3%).	Emulsifying properties	Emulsifying index = 85–90%.	[71]
Chlorophyta, Cyanobacteria	Chlorellaceae, Microcoleaceae	*Chlorella vulgaris*, *Limnospira platensis* (formerly *Arthrospira Platensis*)	Sequential solvent extraction	Total lipids + pigments (% *w*/*w*): *Chlorella*: 11.44 ± 0.131% *Limnospira*: 11.24 ± 0.210% Total carotenoids + chlorophylls: *Limnospira*: 123.93 ± 6.13 mg/100 g *Chlorella*: 99.10 ± 5.45 mg/100 g Protein content: *Limnospira*: 65.38% ± 5.59% *Chlorella*: 53.78% ± 13.51%	Antioxidant	S3: IC_50_ = 90 ± 19.0 μg/mL S2: IC_50_ = 160.2 ± 0.0 μg/mL S5: IC_50_ = 180 ± 10.0 μg/mL C4: IC_50_ = 518 ± 79.0 μg/mL (lowest activity) C3: IC_50_ = 363 ± 73.0 μg/mL C1: IC_50_ = 303 ± 60.0 μg/mL	[72]
Heterokontophyta	Monodopsidaceae	*Nannochloropsis oceanica* 0011NN, *Nannochloropsis * *limnetica* 0065NA	Chloroform–methanol-based biphasic lipid extraction	Total Lipid Content: 23.6 ± 2.6% to 35.3 ± 0.8%. Phospholipids (μg/mg lipid extract): 57.6 ± 8.0 (NL-In) to 104.8 ± 3.3 (NO-Out). Glycolipids (μg/mg lipid extract): 233.2 ± 10.0 (NL-Out) to 274.8 ± 11.0 (NO-In). Neutral lipids + pigments: (22.9 ± 0.8%).	Antioxidant	IC_15_: ranging from 30.4 ±1.8 to 45.7 ± 1.6 μmol	[73]
Haptophyta	Isochrysidaceae	*Chrysotila pseudoroscoffensis*	Folch extraction method: Dichloromethane:Methanol (2:1, *v*/*v*) → Vortexing for 2 min → Repeated centrifugation (626× *g*) → aqueous washing → re-extraction → purification → Drying & quantification.	Moisture: 6.5 ± 0.4%. Ash: 45.5 ± 1.0%. Total Sugars: 11.0 ± 3.2%. Dominant sugars: uronic acids (53.8 mol%), glucose, and galactose. Proteins: 11.6 ± 0.4%. Lipids: 6.4 ± 0.0%.	Antioxidant	IC_50_ (ABTS): 111.9 μg/mL IC_35_ (DPPH): 234.8 μg/mL	[74]
Cyanobacteria	Nostocaceae	*Nostoc commune*	Solvent extraction technique using ethanol: 100 g powder with 300 mL ethanol 1:3 (*w*/*v*) → Room temperature, 1 h → Filtration → Concentration via rotary evaporation → Freeze-drying → Sonication	Total polyphenols: 25.89 ± 1.18 µg Total flavonoids: 19.32 ± 0.45 µg Total terpenoids: 926.53 ± 0.03 µg	Anti-obesity	NEE reduced the body weight (13.5%), fat tissue weight (13.3%), and the serum FFA (19.4%), TG (14.2%), TC (11.8%), and LDL-C (16.4%) of rats.	[75]
Chlorophyta	Chlamydomonadaceae	*Chlamydomonas reinhardtii* (THN6)	NR	NR	Gastrointestinal health	Mice given *C. reinhardtii* biomass: Lost less weight (12%) → weight gain by 2% Humans participants: LowGIS_1g: 85% Rarely Frequency of GIS LowGIS_3g: 73% Rarely Frequency of GIS HighGIS_1g: 57% Often Frequency of GIS HighGIS_3g: 44% Often Frequency of GIS	[76]
Cyanobacteria	Microcoleaceae	*Limnospira platensis* (formerly *Arthrospira platensis*) F&M-C265	NR	NR	Cardiometabolic	Reduced liver weight: 32 gr (*p* < 0.05). Reduced triglycerides: 155 ± 12.62 mg/dL (*p* < 0.05). Reduced total cholesterol: 107 ± 3.45 mg/dL (*p* < 0.01). Reduced systolic and diastolic blood pressure: 136 ± 4.28 and 73 ± 3.92 mm Hg.	[77]
Cyanobacteria	Spirulinaceae	*Limnospira platensis* (formerly *Spirulina platensis*)	NR	Diadinoxanthin (28.01 ± 0.11 μg/g), Alloxanthin/Canthaxanthin (22.76 ± 0.04 μg/g) Diatoxanthin (100.11 ± 0.22 μg/g), Zeaxanthin (113.76 ± 0.15 μg/g), Antheraxanthin (27.20 ± 0.02 μg/g), Echinenone (24.95 ± 0.16 μg/g), β-carotene (1226.99 ± 7.67 μg/g).	Fibromyalgia	Reduced neuroinflammation (astrocyte and microglial activation). Limited oxidative stress (lower MDA, H_2_O_2_, NO; restored SOD, GSH, CAT). Restored biogenic amine levels (NE, DA, 5-HT). Improved behavioral symptoms (pain sensitivity, depression-like behaviors, and locomotion)	[78]
Cyanobacteria, chlorophyta, Heterokontophyta	Microcoleaceae, Chlorellaceae, Phaeodactylaceae	*Limnospira platensis* (formerly *Arthrospira platensis*), *Chlorella vulgaris*, and *Phaeodactylum tricornutum*	Pressurized liquid extraction	*Limnospira*: Magnesium: 383.5 mg, Phosphorus: 752.5 mg, Calcium: 798 mg, Iron: 96.8 mg, Zinc: 2.73 mg, Selenium: 0.11 mg *Chlorella*: Magnesium: 344.3 mg, Phosphorus: 1761.5 mg, Calcium: 593.7 mg, Iron: 259.1 mg, Zinc: 1.19 mg, Selenium: 0.07 mg *P. tricornutum*: Magnesium: 555 mg, Phosphorus: 269 mg, Calcium: 1910 mg, Zinc: 373 mg.	Dietary sup plements	PLE + 100% DMSO, the highest yields *in Limnospira*: Protein: 45 mg/g Polyphenols: 12.5 mg/g Chlorophyll a: 8.0 mg/g Chlorophyll b: 3.0 mg/g Carotenoids: 1.97 mg/g 33 mg/L of Fucoxanthin in *P. tricornutum* 26.50 mg/L of (all-E) β-carotene in *Limnospira* 3.31 mg/L of lutein in *Chlorella*	[79]
Chlorophyta	Scenedesmaceae	*Tetradesmus obliquus* (formerly *Scenedesmus obliquus*)	Ultrasound-assisted cell disruption	Protein content: 40.42%. Insoluble fibers: 16.23% Soluble fibers: 3.14% Phenolic compounds: 1.96% Carotenoids: 1.10% Fatty acids: Oleic acid (C18:1): 1.38% Linoleic acid (C18:2): 0.95% Linolenic acid (C18:3): 0.28%	Hypolipidemic and hypoglycemic	All diets showed > 75% digestibility. Decreased glucose Levels (mg/dL): M100 diet → 128.75 Decreased triglyceride Levels (mg/dL): M50 diet → 75.87 mg/dL	[80]
Cyanobacteria, Heterokontophyta	Microcoleaceae, Fucaceae	*Limnospira platensis* (formerly *Arthrospira platensis*), *Fucus vesiculosus*	Ethanolic extraction using ultrasound-assisted extraction followed by maceration	Total pigment content: 139.91 mg/g Chlorophyll a content: ~28% of total pigments. Highest TPC: *F. vesiculosus* from aquaculture: 11.34 µg GAE/mg *F. vesiculosus* based supplement E: 12.33 µg GAE/mg	Weight Loss	Metabolism of carbohydrates → inhibitory effects: *F. vesiculosus* + *Limnospira* supplement F: IC_25_ = 4.54 ± 0.81 µg/mL *Limnospira*-Supplement B: IC_25_= 10.17 ± 0.95 µg/mL *F. vesiculosus* (Wild): IC_25_= 30.59 ± 0.08 µg/mL Maximum aldose reductase activity: *F. vesiculosus* supplement E: IC_25_= 72.39 ± 9.88 µg/mL	[81]
Chlorophyta	Chlorellaceae, Microcoleaceae	*Chlorella vulgaris*	NR	NR	Oxidative stress and antioxidant genes expression in liver and ovaries	▸In liver tissue: Mean MDA = 6.61 ± 1.54 nmol/mg (*p* < 0.001) Mean SOD = 169.70 ± 18.3 U/mg (*p* < 0.002) Mean CAT = 8.70 ± 0.30 U/mg (*p* < 0.010) Mean GSH = 9.71 ± 0.49 μmol/g (*p* < 0.001) ▸In uterus tissue: Mean MDA = 1.70 ± 0.46 nmol/mg (*p* < 0.001) Mean TAC = 244.00 ± 18.00 μmol/mg (*p* < 0.012) Mean SOD = 144.40 ± 18.4 U/mg (*p* < 0.002) Mean CAT = 3.87 ± 0.02 U/mg (*p* < 0.001) Mean GSH = 9.52 ± 0.48 μmol/g (*p* < 0.001) ▸Gene expression: In liver: sod1: 2.02 ± 0.35 fold change (*p* < 0.009) gpx1: 5.89 ± 0.76 fold change (*p* < 0.004) ▸In ovaries: sod1: 1.76 ± 0.61 fold change (not significant, *p* < 0.42) gpx1: 2.07 ± 0.39 fold change (*p* < 0.030)	[82]
Cyanobacteria	Spirulinaceae	*Limnospira platensis* (formerly *Spirulina platensis*)	Solvent extraction using DMSO	NR	Functional food additive	▸High-affinity Binding: Binding constant (Ka) = 2 × 10^6^ M^−1^ ▸Improved Thermal Stability: The BSA–PCB complex shows increased thermal stability compared to free BSA or PCB.	[83]
Cyanobacteria	Microcoleaceae	*Limnospira platensis* (formerly *Arthrospira platensis*)	Ultrasound-assisted extraction using chloroform	NR	Obesity and intestinal diseases	Hypercaloric diet: Emax dropped to 32.7%, pEC_50_: increased to 6.6. *A. platensis* (25 mg/kg) in HCD: improved Emax = 63.9% and pEC_50_ = 6.2. With ROCK inhibitor (Y27632): HCD + *A. platensis*: Emax = 49.1%, pEC_50_ = 5.6. With NOS inhibitor (L-NAME): HCD + *A. platensis*: Emax = 34.1%, pEC_50_ = 4.6. With COX inhibitor (Indomethacin): HCD + *A. platensis*: Emax = 54.4% With NOX inhibitor (Apocynin): HCD + *A. platensis*: Emax = 40.9%, pEC_50_ = 4.8.	[84]
Cyanobacteria	Spirulinaceae	*Limnospira (formerly Spirulina* LEB-18)	Methanol-based solvent extraction	Caffeic acid: 47.02 µg/g extract Hydroxybenzoic acid: 54.66 µg/g extract Chlorogenic acid: 19.27 µg/g extract Gallic acid: 17.74 µg/g extract Protocatechuic acid: 11.06 µg/g extract	Gastrointestinal conditions	Initial size of lactoferrin-loaded liposomes: ▸After 1 min in simulated intestinal fluid (SIF): 2389 ± 215 nm (PDI = 0.69 ± 0.11) ▸After digestion in gastric fluid without pepsin: reduced to ~500 nm Initial ζ-potential (LF-loaded liposomes): +13.0 ± 0.4 mV In SGF: ▸Without pepsin: +9.2 ± 1.0 mV ▸With pepsin: 0 mV In SIF: ▸Without pancreatic lipase: –44.2 ± 0.4 mV	[85]
Cyanobacteria	Chroococcaceae, Microcoleaceae, Leptolyngbyaceae, Pseudanabaenaceae, Microcoleaceae, Spirulinaceae, Leptolyngbyaceae, Nodulariaceae, Aphanizomenonaceae	*Chroococcus Chroo-CH*, *Limnospira platensis* (formerly *Arthrospira platensis*) *Arthro*-KB, *Leptolyngbya Osci*-NB-01, *Leptolyngbya Lepto*-CH, *Limnothrix Osci*-BM-01, *Planktothrix agardhii* Plank-SS-01, *Limnospira (formerly Spirulina Spir*-ML), *Lyngbya Lyng*-ML, *Anabaenopsis circularis* Pseud-01, *Raphidiopsis raciborskii* (formerly *Cylindrospermopsis raciborskii*) Cyl-NB-05.	Fatty acids: solvent mixture of dichloromethane: methanol:0.7% NaCl (1:2:0.8, *v*/*v*/*v*). Proteins: Manual grinding of 50 mg of freeze-dried sample + 4 mL of distilled water → centrifugation. Carbohydrate: extraction/hydrolysis method: Acid hydrolysis of 100 mg dry biomass using 2.5 N HCl at 100 °C for 1 h. Phycobiliproteins: Tris-Cl buffer (pH 8.1) → Freezing/thawing cycles and sonication → Centrifugation at 12,000 rpm.	Protein Content (% DW) → Osci-BM-01: ~30%-Cyl-NB-05: ~30%-Chroo-CH: <5%. High polysaccharide → Lepto-CH: 46.6% DW-Cyl-NB-05: 33.3% DW-Chroo-CH: 30.6% DW-Other strains: <30% DW. Total phycobiliproteins max value: Cyl-NB-05: >3150 µg/mL PC: 2873 µg/mL APC + PE: 277 µg/mL PC across strains: 11 to 671 µg/mL	NR	▸linolenic acid, (C18:3): 25%. ▸Plank-SS-01, Lepto-CH and Osci-NB 01, contained a high percentage of oleic acid (25%, 28%, and 27%, respectively). ▸Cylind-NB was the most productive of total phycobiliproteins = 3000 µg/mL.	[86]
Heterokontophyta, Chlorophyta, Heterokontophyta	Monodopsidaceae, Chlorellaceae, Phaeodactylaceae	*Nannochloropsis oceanica*, *Chlorella vulgaris*, *Phaeodactylum tricornutum*	NR	Crude protein [g/kg dry mass]: *N. oceanica* = 387/*P. tricornutum* = 429/*C. vulgaris* = 542. Crude lipids [g/kg dry mass]: *N. oceanica* = 167/*P. tricornutum* = 99/*C. vulgaris* = 104. Crude ash [g/kg dry mass]: *N. oceanica* = 237/*P. tricornutum* = 177/*C. vulgaris* = 71. Calcium [g/kg dry mass]: *N. oceanica* = 2.6/*P. tricornutum* = 24.1/*C. vulgaris* = 3.4. Phosphorus [g/kg dry mass]: *N. oceanica* = 11.9/*P. tricornutum* = 27.9/*C. vulgaris* = 14.0. Caloric value [MJ/kg dry mass]: *N. oceanica* = 22.0/*P. tricornutum* = 19.2/*C. vulgaris* = 22.6.	Metabolic conditions	Energy content in WSD group: 18.6 MJ/kg (4.4 kcal/g) feed. Caloric intake in the WSD groups: 13.6% (*p* < 0.01). *N. oceanica*: significant reduced liver steatosis in mice fed a WSD (from 17% to 4.7%, *p* < 0.002).	[87]
Haptophyta	Isochrysidaceae	*Tisochrysis lutea*	Fucoxanthin: Freeze-dried biomass treated with methanol, diethyl ether/petroleum ether, and NaCl aqueous solution (liquid–liquid extraction) → Evaporation, then resuspension in methanol/MTBE (4:1).	Proteins: 42.4% Fibers: 18.2% Ash: 13.1% Total Lipids: 7.9% (MUFAS: 3.8%/PUFAS: 4.1%) Carotenoids: 0.6%	Dietary safety and tolerability	Digestibility: lower than AIN-76 diet (−4%). Daily water intake: Doubled compared to controls (NaCl content: 1.5% vs. 0.3%). Urinary sodium excretion: 5.7 ± 0.45 mEq/24 h Relative heart weight: Increased (*p* < 0.05) Uric acid excretion: 0.15 ± 0.01 mg/24 h Liver weight: reduced (*p* < 0.05). Total cholesterol: Increased HDL cholesterol: +112% (*p* < 0.05) Fecal lipid excretion: +75% Plasma triglycerides: −73% (*p* = 0.06) Fucoxanthin in diet: 0.12% DHA intake (rats): ~30 mg/day ω-6/ω-3 ratio: 0.58 (favorable)	[88]
Cyanobacteria, Heterokontophyta	Microcoleaceae, Monodopsidaceae	*Limnospira* sp. *(formerly Arthrospira* sp.), *Nannochloropsis* sp.	High-pressure homogenisation: Biomass is rehydrated in distilled water at 8% (*w*/*v*) → Mechanical Pre-treatment: The suspension is mixed at 5000 rpm for 5 min → high-pressure homogenisation at 300, 600, and 900 bar.	Chlorophyll a (mg/mL): *Limnospira* = 0.12–0.36/*Nannochloropsis* = 0.11–0.24. Carotenoides (mg/mL): *Limnospira* = 0.19–0.29/*Nannochloropsis* = 0.10–0.17. Total phenolic content (mg GAE/g DM): *Limnospira* = 240.13–247.78 *Nannochloropsis* = 195.63–225.82 Protein content: *Limnospira* = 29–31% *Nannochloropsis* = 3.8–8.7%	Bioprotective potential	Digestibility: *Limnospira* and *Nannochloropsis* → Enhanced post-pancreatin FRAP: *Limnospira* → Unaffected by HPH *Nannochloropsis* → Improved at 900 bar only DPPH (%): *Limnospira* → ~46–48% *Nannochloropsis* → ~50–56% Bioprotective effect: *Limnospira* → best at 600 bar Cytotoxicity (>48 h): *Limnospira* and *Nannochloropsis* → Observed at ≥6 mg/mL	[89]
Cyanobacteria	Spirulinaceae	*Limnospira maxima* (formerly *Spirulina maxima*) UTEX LB2342	Total fat was extracted using the Soxhlet method.	Proteins: 59.0% Carbohydrates: 19.11% Lipids: 3.12% Crude fiber: 3.05% Moisture: 7.42% Ash: 8.22%	Microcapsules for nutritional enrichment preventing toxicity	Significant improvement in: Testosterone: 3.69 ± 0.21 a ng/mL LH: 1.62 ± 0.54 ng/L FSH: 2.58 ± 0.51 ng/L TNF-α reduction: 59.32 ± 1.41 pg/mL Decrease in: MDA: 2.74 ± 0.41 nmol/mg ACP: 12.03 ± 0.84 U/g LDH: 93.11 ± 1.53 U/g	[2]
Cyanobacteria	Microcoleaceae	*Limnospira platensis* (formerly *Arthrospira platensis*)	NR	Fermentation Time TPC (mg GAE/g dw) 36 h 17.87 ± 0.77 Fermentation Time C-Phycocyanin (mg/g dw) 36 h 187.00 ± 3.79	functional ingredient in nutraceuticals	Total phenolic content increased by 112% after 36 h. FRAP: improved by 85% at 36 h. ORAC: rose by 36% at 36 h. DPPH: 60% increase after 24 h. FMC increased by 94% after 72 h.	[90]
Cyanobacteria	Spirulinaceae	*Limnospira platensis* (formerly *Spirulina platensis*)	Sequential solvent extraction: Extraction with 95% ethanol 1:10 (*w*/*v*), 45 °C, 0.5 h → Filtrate 1 →Extraction with 55% ethanol → 1:10 (*w*/*v*), 45 °C, 0.5 h → Filtrate 2 →Extraction with water → 1:10 (*w*/*v*), 45 °C, 0.5 h → Filtrate 3	NR	Lipid metabolism	Improved lipid profiles in HFD-fed rats over 8 weeks by: Lowering TG (up to 33.33%) Reducing TC (up to 27.62%) Elevating HDL-C (by 24.44%) Decreasing LDL-C significantly (*p* < 0.01)	[91]
Cyanobacteria	Spirulinaceae	*Limnospira platensis* (formerly *Spirulina platensis*)	NR	Crude protein, ash, lipid, carbohydrate, sugar, fiber, chlorophyll, phycocyanin, calcium, chromium, sodium, magnesium, gamma-linolenic acid, vitamin B12, vitamin D, total carotenoids (β-carotene).	Supplementation for an improved bone strength and stiffness.	Improved Tb.N (1/mm): 3.39 ± 0.69 Increased number of expressed OCN genes: 2.00 of relative expression.	[92]
Charophyta	Desmidiaceae, Spirogyraceae	*Cosmarium blytii* BEA0204, *Cosmarium* sp. BEA0208, *Spyrogyra* sp. BEA0666B	Ultrasound-assisted methanolic extraction	Carbohydrate content (% DW): *Cosmarium* sp. → 43.2% *Cosmarium blytii* → 41.8% *Spirogyra* sp → 35.9% Glutamic Acid (mg/g): *Cosmarium* sp. → 12 *Cosmarium blytii* → < 12 *Spirogyra* sp → < *Cosmarium* sp.	Functional Food and Feed Uses	Carbohydrate content: 35.8% to 43.3%. Glutamic acid: 3.63 to 12.2 mg/g. Highest in *Cosmarium blytii*: 24.02 mg/g. DPPH Assay: 21.2%	[93]
Cyanobacteria	Microcoleaceae	*Limnospira platensis* (formerly *Arthrospira platensis*)	Cold aqueous extraction	NR	Vegan emulsion	DPPH: 15.4 ± 0.16 to 43.0 ± 1.5 µmol TE/g FRAP: 169.38 ± 5.75 to 343.91 ± 21.12 µmol Fe^2+^/g	[94]
Chlorophyta	Dunaliellaceae, Chlorellales incertae sedis	*Dunaliella salina* C5, *Dunaliella salina* C13, *Picochlorum* spp HM1	Treatment 1 (T1): Cold Ethanolic Extraction Treatment 2 (T2): Forced Cold Ethanolic Extraction Treatment 3 (T3): Forced Aqueous Extraction Treatment 4 (T4): Forced Aqueous Extraction	Protein (g/100 g DM) *D. salina* C13: 57.0 Fat (g/100 g DM) *D. salina* C13: 6.96 Carbohydrates (g/100 g DM) *Picochlorum* spp. HM1: 32.6 Ash (g/100 g DM) *D. salina* C13: 22.6 Total-P (mg/g ash) *Picochlorum* spp. HM1: 70.4 Energy (Kcal/100 g) *Picochlorum* spp. HM1: 366.3 Energy (Kjul/100 g) *Picochlorum* spp. HM1: 1531.0	Nutrition and diet	ABTS (T4) extracts (highest in *Picochlorum* spp. HM1): 6928.4 ± 138.4 µg GAE/g	[95]
Chlorophyta	Dunaliellaceae	*Dunaliella salina* (IBRC-M 50030), *Dunaliella viridis* (IBRC-M 50069), *Dunaliella* spp. (IBRC-M 50065).	Protein: solubilization in phosphate buffer with polyvinylpyrrolidone Carbohydrate: Phenol-sulfuric acid method Dietary Fiber: Van Soest method Total Fat Content: Soxhlet extraction using petroleum ether Fatty Acid Profile: Bligh and Dyer method Pigment Content (Chlorophylls & Carotenoids): Biomass centrifuged and extracted with acetone Total Phenolic Compounds: 80% methanol extraction at 25 °C for 10 min → Mixed with Folin–Ciocalteu reagent and sodium carbonate	Protein: *D. salina*: 22.50 ± 0.22 *Dunaliella* sp.: 17.68 ± 0.11 *D. viridis*: 19.67 ± 0.15 Carbohydrate (mono- and disaccharide): *D. salina*: 12.37 ± 0.26 *Dunaliella* sp.: 7.59 ± 0.39 *D. viridis*: 10.26 ± 0.13 Lipid: *D. salina*: 13.19 ± 0.26 *Dunaliella* sp.: 25.02 ± 0.15 *D. viridis*: 20.80 ± 0.45 Dietary fiber: *D. salina*: 48.82 ± 0.08 *Dunaliella* sp.: 48.16 ± 0.13 *D. viridis*: 42.10 ± 0.14 Total phenolics (mg/100 g): *D. salina:* 1.87 ± 0.01 *Dunaliella* sp.: 1.68 ± 0.09 *D. viridis*: 2.42 ± 0.02	Nutraceuticals and food	High pigment content in *D. salina* (~11.5%). DPPH: 55.63% of inhibition. High levels of polyunsaturated fatty acids: EPA (11.26%) and DHA (6.15%).	[96]
Chlorophyta	Scenedesmaceae	BGLR8, BGLR16	Carbohydrates: Solvent-assisted thermal extraction Proteins: Alkaline thermal extraction Lipids: Solvent + ultrasonic extraction Carotenoids: Cold solvent extraction Flavonoids: Solvent extraction with complexing agents Astaxanthin: Acid hydrolysis + solvent extraction Phycocyanin: Inorganic acid extraction β-Carotene: Organic solvent extraction Total Phenols: Solvent extraction + redox assay	Carbohydrates BGLR16: 191 mg/g DW BGLR8: 124 mg/g DW Proteins BGLR16: 538 mg/g DW (53.8%) BGLR8: 511 mg/g DW (51.1%) Lipids BGLR16: >2% DW BGLR8: 86 mg/g DW (8.6%) Phenolic Compounds BGLR16: 3.62 mg/g DW BGLR8: 4.46 mg/g DW	Nutraceuticals and bioactive components	BGLR8 outperformed BGLR16 in terms of: Lipids: 86 mg/g Total chlorophyll: 29.42 mg/g Carotenoids: 28.82 mg/g Phenols: 4.46 mg/g Phycocyanin: 52 mg/g Astaxanthin: 19.27 mg/g β-Carotene: 5.6 mg/g Antioxidant activity: 31.73%	[97]

NR: Not reported or not specified in the original study.

## Data Availability

No new data were created or analyzed in this study. Data sharing is not applicable to this article.
